# Antioxidant Benzophenones and Biphenyls from an Endophytic Fungus *Penicillium* sp. QM-4

**DOI:** 10.3390/molecules31152720

**Published:** 2026-08-05

**Authors:** Chuan-Mao Zhang, Xiang-Gui Lei, Jun Ma, Jin-Feng Cao, Yang Xiao, Jian-Hai Ding

**Affiliations:** Key Laboratory of Green Catalytic Materials and Technology of Ningxia Province, College of Chemistry and Chemical Engineering, Ningxia Normal University, Guyuan 756099, China; 15090585657@163.com (C.-M.Z.); leixianggui@nxnu.edu.cn (X.-G.L.); majun_0699@nxnu.edu.cn (J.M.); 82018003@nxnu.edu.cn (J.-F.C.); 18645409819@163.com (Y.X.)

**Keywords:** *Penicillium* sp. QM-4, benzophenone, biphenyl, antioxidant activity

## Abstract

The endophytic fungi from plants are recognized as a valuable source of structurally diverse secondary metabolites with intriguing bioactivities. Three previously undescribed compounds, including two benzophenones, 2-(2,6-dihydroxy-4-methylbenzoyl)benzoic acid (**1**) and 2-(2,6-dihydroxy-4-(hydroxymethyl)benzoyl)benzoic acid (**2**), one biphenyl, 4′,5,5′-trihydroxy-3-methoxy-2′-methyl-(1,1′-biphenyl)-2-carbaldehyde (**4**), along with two known compounds (**3** and **5**), were isolated from the endophytic fungus *Penicillium* sp. QM-4 associated with *Pteris cretica*. Their structures were established by extensive spectroscopic analysis of HRESIMS, UV, IR, and 1D and 2D NMR data. Free radical scavenging activity of compounds **1**–**5** was assessed, and compound **3** displayed a significant antioxidant effect in the DPPH assay. Structure–activity relationships of compounds **1**–**3** were also discussed. These findings enrich the structural diversity and bioactivities of benzophenones and biphenyls derived from fungal source.

## 1. Introduction

Benzophenones and biphenyls are important medicinally fungal metabolites [1,2,3,4,5,6,7]. These metabolites are linked with -OH groups and exhibit promising antioxidant activity. The endophytic fungi from plants possess considerable species abundance and diversity, which are increasingly recognized as a distinctive source of structurally diverse and intriguing bioactive natural products [8]. *Penicillium* represents one of the largest fungal genera present in many plants. The *Penicillium* genus produce various secondary metabolites including polyketides, alkaloids, terpenoids, and benzophenones [9,10]. These compounds exhibit promising biological activities, such as cytotoxic, antibacterial, and antioxidant effects. Sulochrin, a benzophenone isolated from the marine mangrove-derived fungus *Penicillium citrinum*, revealed protection efficacy on H_2_O_2_-induced oxidative injury on PC12 cells (% viability 51.66) and scavenged DPPH radicals (IC_50_ 18.90 mM) [11].

*Penicillium* sp. QM-4 is an endophytic fungus isolated from the traditional Chinese medicinal plant *Pteris cretica* in Kunming, Yunnan. During our continuing search for bioactive compounds with interesting structures from endophytic fungi in medicinal or edible plants, chemical investigation of this strain *Penicillium* sp. QM-4 yielded three previously undescribed compounds, including two benzophenones, 2-(2,6-dihydroxy-4-methylbenzoyl)benzoic acid (**1**) and 2-(2,6-dihydroxy-4-(hydroxymethyl)benzoyl)benzoic acid (**2**), one biphenyl, 4′,5,5′-trihydroxy-3-methoxy-2′-methyl-(1,1′-biphenyl)-2-carbaldehyde (**4**), together with two known compounds (**3** and **5**). Herein, we reported their isolation, structural elucidation, as well as their antioxidant activity against free radicals.

## 2. Results and Discussion

**Figure 1:**
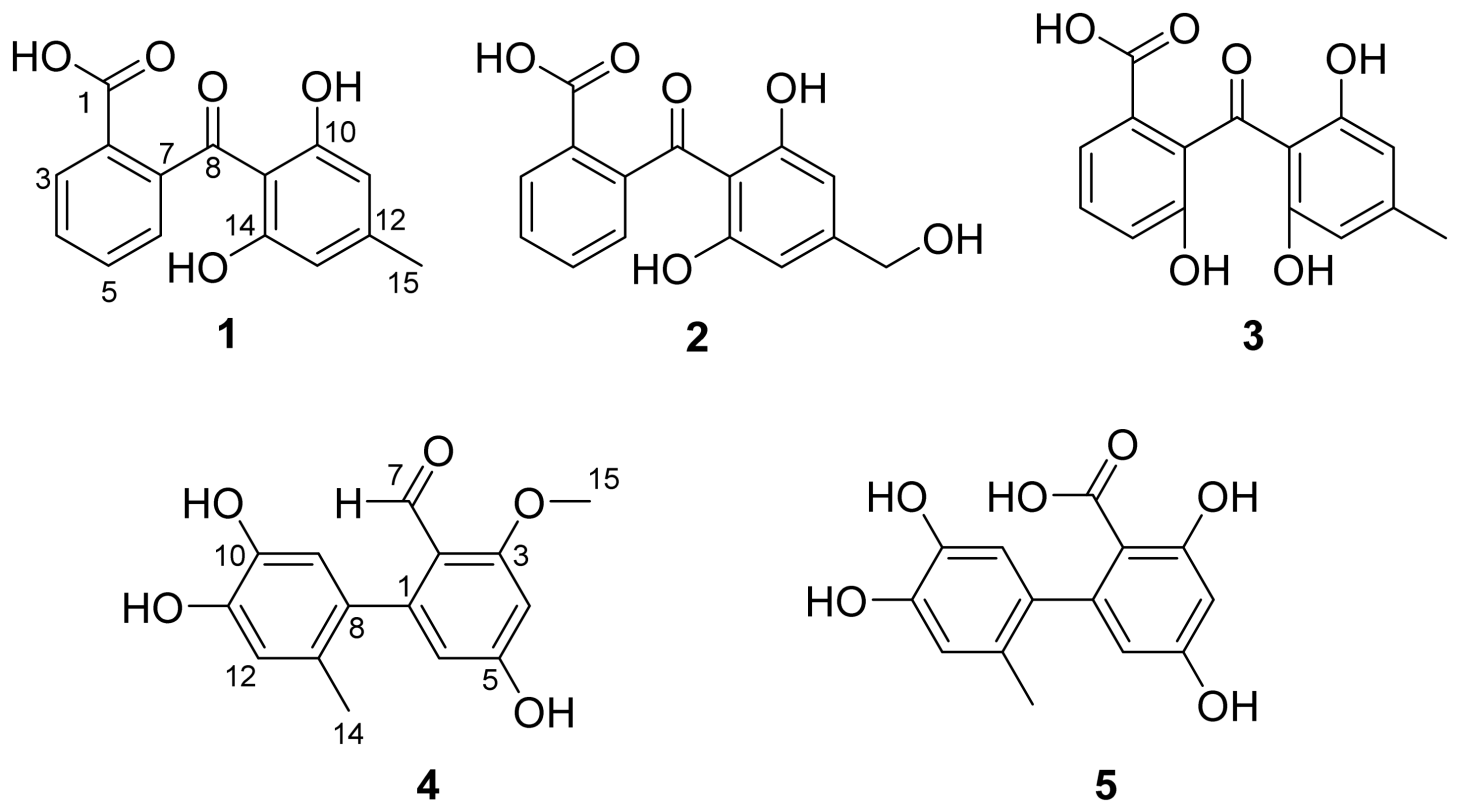
Structures of compounds **1**–**5**.

**Figure 2:**
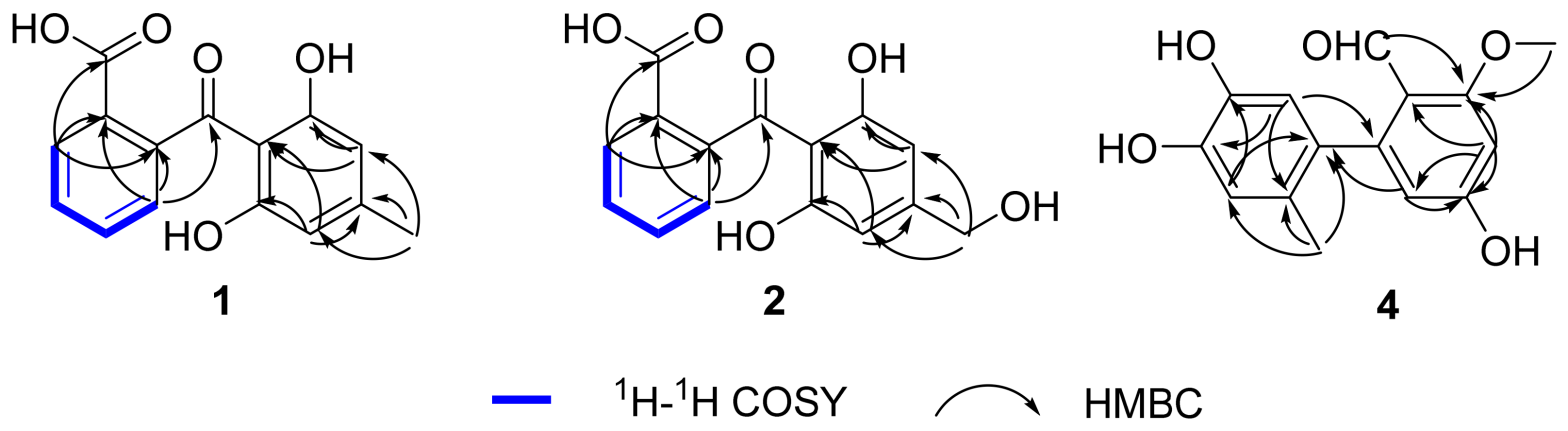
Key ^1^H-^1^H COSY and HMBC correlations of **1**, **2**, **4**.

Compound **1** (Figure 1) was obtained as brown oil. Its molecular formula was determined as C_15_H_12_O_5_ based on HR-ESI-MS *m*/*z* 295.0584 [M + Na]^+^ (calculated for 295.0577), corresponding to ten degrees of unsaturation. The IR spectrum exhibited absorption bands indicating the presence of hydroxyl (3438 cm^−1^) and carbonyl (1636 cm^−1^). The UV spectrum showed absorption bands at *λ*_max_ 337, 283, and 204 nm indicating the presence of a benzophenone chromophore. The ^1^H NMR of compound **1** (Table 1) exhibited six aromatic methine hydrogen signals at *δ*_H_ 7.99 (d, *J* = 7.7 Hz, 1H, H-3), 7.48 (dd, *J* = 7.7, 7.6 Hz, 1H, H-4), 7.59 (dd, *J =* 7.7, 7.6 Hz, 1H, H-5), 7.19 (d, *J* = 7.6 Hz, 1H, H-6), 6.16 (s, 2H, H-11, H-13), and one methyl signal at *δ*_H_ 2.21 (s, 3H, H-15). Based on ^13^C NMR (Table 1) and HSQC spectroscopic data, the fifteen ^13^C resonances were twelve aromatic carbons at *δ*_C_ 128.4 (C-2), 129.2 (C-3), 127.8 (C-4), 131.7 (C-5), 125.1 (C-6), 145.7 (C-7), 108.2 (C-9), 161.9 (C-10), 107.5 (C-11/13), 148.6 (C-12), 161.9 (C-14), one carboxylic carbon at *δ*_C_ 168.6 (C-1), one ketone carbon at *δ*_C_ 202.4 (C-8), and one methyl carbon at *δ*_C_ 20.7 (C-15). The above NMR spectra of compound **1** were like those of cephalanone F (**3**) [12], except for the absence of a hydroxy at C-6. The key difference was supported by ^1^H-^1^H COSY (Figure 2) correlations of H-3/H-4/H-5/H-6. Thus, the structure of compound **1** was determined and named as 2-(2,6-dihydroxy-4-methylbenzoyl)benzoic acid.

HR-ESI-MS (observed at *m*/*z* 289.0711, calcd at *m*/*z* 289.0707 [M + H]^+^) analysis of compound **2** (Figure 1) demonstrated that it had the molecular formula of C_15_H_12_O_6_. The IR, UV, and NMR spectra (Table 1) showed that compounds **2** and **1** were similar, with the only differences of the hydroxymethyl at C-15 in **2** replacing the methyl group in **1**. This assumption was corroborated by the key HMBC (Figure 2) correlations from H_2_-15 to C-11, C-12, and C-13. Finally, the structure of **2** was determined and named as 2-(2,6-dihydroxy-4-(hydroxymethyl)benzoyl)benzoic acid.

Compound **4** (Figure 1) was isolated as a yellow oil. Its molecular formula C_16_H_16_O_5_ was determined by the HRESIMS at *m*/*z* 275.0922 [M + H]^+^ (calculated for C_16_H_17_O_5_^+^ *m*/*z* 275.0914), indicating nine degrees of unsaturation. The UV spectrum absorption bands at *λ*_max_ 295, 240, and 215 nm and IR absorption bands at 3438 cm^−1^ (hydroxyl) and 1636 cm^−1^ (carbonyl) suggested the presence of a biphenyl chromophore. The ^1^H NMR spectrum of **4** (Table 2) exhibited proton resonances corresponding to one aldehyde hydrogen signal at *δ*_H_ 10.45 (s, 1H), four aromatic methine hydrogen signals at *δ*_H_ 6.44 (d, *J* = 2.6 Hz, 1H), 6.18 (d, *J* = 2.6 Hz, 1H), 6.49 (s, 1H), 6.59 (s, 1H), one methoxy hydrogen signal at *δ*_H_ 3.82 (s, 3H) and one methyl hydrogen signal at *δ*_H_ 1.92 (s, 3H). The ^13^C NMR (Table 2) and HSQC spectra of **4** showed 16 carbon resonances, including twelve aromatic carbons at *δ*_C_ 146.7 (C-1), 105.7 (C-2), 163.6 (C-3), 99.2 (C-4), 161.9 (C-5), 110.0 (C-6), 134.0 (C-8), 115.2 (C-9), 141.9 (C-10), 143.6 (C-11), 115.9 (C-12), 125.9 (C-13), one aldehyde carbon signal at *δ*_C_ 195.7 (C-7), one methoxy carbon signal at *δ*_C_ 54.5 (C-15), and one methyl carbon signal at *δ*_C_ 17.9 (C-14). The above NMR spectra of compound **4** were like those of desmethylaltenusin (**5**) [13], with the differences of the aldehyde at C-2 and methoxy at C-3 in **4** replacing the carboxyl and hydroxy in **5**, respectively. This assumption was corroborated by the key HMBC correlations from H-7, and H_3_-15 to C-3 (Figure 2). Thus, the structure of compound **4** was determined and named as 4′,5,5′-trihydroxy-3-methoxy-2′-methyl-(1,1′-biphenyl)-2-carbaldehyde.

In addition to the three undescribed compounds, two known compounds were purified: cephalanone F (**3**) [12] and desmethylaltenusin (**5**) [13]. All aforementioned compounds were identified by comparing their NMR spectroscopic data with literature-reported values.

Compounds **1**–**5** were evaluated for their antioxidant activities, as summarized in Table 3. Compound **3** displayed a significant antioxidant effect in the DPPH assay. The additional hydroxyl group at C-6 in compound **3** compared to compound **1** significantly enhanced DPPH radical scavenging activity [1].

## 3. Experimental Section

### 3.1. General Experimental Procedures

Optical rotations were recorded on an Autopol VI digital polarimeter (Rudolph, Hackettstown, NJ, USA). UV data were obtained on a Shimadzu UV–2401PC spectrophotometer (Shimadzu, Kyoto, Japan). Infrared spectroscopy (IR) spectra were obtained on a Bruker Tensor 27 FT–IR spectrometer (Bruker Corporation, Karlsruhe, Germany) with KBr pellets. Nuclear Magnetic Resonance (NMR) spectra were obtained on a Bruker AV–400 instrument (Bruker Corporation, Karlsruhe, Germany) with tetramethylsilane (TMS) as the internal standard at room temperature. Mass spectrometry data were recorded on an AB Sciex X500R QTOF instrument (AB Sciex, Marlborough, MA, USA). Semi-preparative reversed-phase C18 high-performance liquid chromatography (Agilent 1260 Infinity II Prime, Agilent Technologies, Santa Clara, CA, USA) was employed. Silica gel (200–300 mesh, Qingdao Marine Chemical Ltd., Qingdao, China) and Sephadex LH–20 (Amersham Biosciences, Uppsala, Sweden) were used for open column chromatography (CC). Fractions were monitored by TLC. Spots were visualized by heating silica gel plates immersed in vanillin–H_2_SO_4_ in ethanol.

### 3.2. Fungal Material

The fresh plant sample *Pteris cretica* was collected at Changchong Mountain, Kunming, Yunnan Province, China, in July 2022. The plant sample was washed in 75%.

EtOH and sterile distilled H_2_O, and the surface-sterilized sample was chopped into small pieces and then inoculated in potato dextrose agar (PDA) plates at 28 °C. After cultivation at 28 °C for 3–5 days, the mycelium was purified according to colony morphology. Genomic DNA was extracted using a fungal genomic DNA rapid extraction kit (Qingke Biotech, Kunming, China). PCR amplification with the primers ITS4 and ITS5 was performed using the fungal genomic DNA as template, followed by Sanger sequencing. The sequence data was submitted to the GenBank (Accession No. NR11820.1). The sequencing results were compared against the NCBI database, confirming the identity of the fungus as *Penicillium* sp. The fungus named *Penicillium* sp. QM-4 was deposited at the Laboratory of Natural Medicinal Chemistry, College of Chemistry and Chemical Engineering, Ningxia Normal University, Guyuan, People’s Republic of China.

The culture medium consisted of rice (14.40 kg) and water (18.04 L), sterilized by autoclaving at 121 °C for 25 min. After cooling, the fungal suspension was inoculated, followed by 29-day fermentation.

### 3.3. Extraction and Isolation

The fermented product was extracted with methanol four times. The combined extracts were suspended in water, then subjected to ethyl acetate extraction and vacuum concentration to give crude extract (70.2 g). Subsequently, the extract was preliminarily separated by normal-phase silica gel column chromatography with a gradient elution of CH_2_Cl_2_-MeOH (100:0–10:90), affording six fractions designated Fr.1–Fr.6.

Fr.2 was subjected to silica gel CC with CH_2_Cl_2_-MeOH (100:0–40:1), yielding Fr.2.1–2.3. Fr.2.3 was purified by Sephadex LH-20 CC (CH_2_Cl_2_-MeOH, 1:1) followed by semipreparative HPLC (MeOH-H_2_O-H_3_PO_4_, 44.8:55.0:0.2–99.8:0:0.2, 3 mL/min) to give compound **1** (8.8 mg, t*_R_* = 11.40 min).

Fr.3 was subjected to silica gel CC with CH_2_Cl_2_-MeOH (100:0–40:1), yielding Fr.3.1–3.3. Fr.3.2 was eluted by silica gel column chromatography (CH_2_Cl_2_-MeOH, 100:1–30:1) and further purified by Sephadex LH-20 CC (CH_2_Cl_2_-MeOH, 1:1) to afford compound **4** (3.5 mg). Combined Fr.3.2.2-Fr.3.2.4 were separated by Sephadex LH-20 CC (CH_2_Cl_2_-MeOH, 1:1), affording compound **2** (37.1 mg).

Fr.4 was subjected to silica gel CC with CH_2_Cl_2_-MeOH (70:0–20:1), yielding Fr.4.1–4.3. Fr.4.1 was subjected to silica gel CC with CH_2_Cl_2_-MeOH (100:0–30:1) and further purified by Sephadex LH-20 CC (CH_2_Cl_2_-MeOH, 1:1) to afford compound **3** (9.9 mg).

Fr.5 was subjected to silica gel CC with CH_2_Cl_2_-MeOH (80:0–15:1), yielding Fr.5.1–5.4. Combined Fr.5.2 and Fr.5.3 were separated by Sephadex LH-20 CC (CH_2_Cl_2_-MeOH, 1:1) to afford compound **5** (9.5 mg).

### 3.4. Characterization Data

#### 3.4.1. 2-(2,6-Dihydroxy-4-methylbenzoyl)benzoic Acid (**1**)

Brown oil; C_15_H_12_O_5_; UV (MeOH) *λ*_max_ (log *ε*): 204 (3.52), 283 (3.11), 337 (2.41) nm; IR (KBr) *ν*_max_: 3438, 1636, 1487, 1447, 1385, 927 cm^−1^; ^1^H (400 MHz, CD_3_OD) and ^13^C NMR (100 MHz, CD_3_OD) NMR data, Table 1; HR-ESI-MS: *m*/*z* 295.0584 [M + Na]^+^(calcd for C_15_H_12_O_5_Na, 295.0577).

#### 3.4.2. 2-(2,6-Dihydroxy-4-(hydroxymethyl)benzoyl)benzoic Acid (**2**)

Brown oil; C_15_H_12_O_6_; UV (MeOH) *λ*_max_ (log *ε*): 206 (3.95), 281 (3.48), 339 (2.85) nm; IR (KBr) *ν*_max_: 3437, 1635, 1491, 1432, 1379, 927 cm^−1^; ^1^H (400 MHz, CD_3_OD) and ^13^C NMR (100 MHz, CD_3_OD) NMR data, Table 1; HR-ESI-MS: *m*/*z* 289.0711 [M + H]^+^(calcd for C_15_H_13_O_6_, 289.0707).

#### 3.4.3. 4′,5,5′-Trihydroxy-3-methoxy-2′-methyl-(1,1′-biphenyl)-2-carbaldehyde (**4**)

Yellow oil; C_16_H_16_O_6_; ${\left[\alpha \right]}_{D}^{20}$-5.5 (*c* = 0.04, MeOH); UV (MeOH) *λ*_max_ (log *ε*): 215 (3.32), 240 (3.14), 295 (2.98) nm; IR (KBr) *ν*_max_: 3440, 1627, 1491, 1438, 1383, 1249, 1164 cm^−1^; ^1^H (400 MHz, CD_3_OD) and ^13^C NMR (100 MHz, CD_3_OD) NMR data, Table 2; HR-ESI-MS: *m*/*z* 289.0711 [M + H]^+^(calcd for C_15_H_13_O_6_, 289.0707).

### 3.5. Antioxidant Assays

DPPH radical scavenging activity was determined by the reported method [14]. DPPH was accurately weighed and dissolved in ethanol to prepare 0.5 mol/L DPPH storage solution, which was refrigerated away from light. The 100 μL DPPH solution was added to a 96-well plate, and then a series of sample solutions and a positive control vitamin C (V_C_) solution were added. The reaction lasted for 30 min at room temperature, avoiding light. After the reaction was completed, the absorbance values were determined at 517 nm (three wells were set for each sample).

ABTS radical scavenging activity was determined according to the reported method [15]. The ABTS solution was diluted with ethanol to an absorbance of about 0.90 at 734 nm. Subsequently, 100 μL of the compounds were added to 100 μL of ABTS solution and reacted with light protection for 10 min at room temperature. Finally, the absorbance values were determined at 734 nm. DMSO and V_C_ were used as a negative and positive control, respectively.

Data processing and IC_50_ calculation for each compound were performed with SPSS (IBM SPSS Statistics 32), and the corresponding data are listed in (Table 3).

## 4. Conclusions

In summary, three previously undescribed benzophenones, 2-(2,6-dihydroxy-4-methylbenzoyl)benzoic acid (**1**) and 2-(2,6-dihydroxy-4-(hydroxymethyl)benzoyl)benzoic acid (**2**), and biphenyl 4′,5,5′-trihydroxy-3-methoxy-2′-methyl-(1,1′-biphenyl)-2-carbaldehyde (**4**), along with two known compounds (**3** and **5**), were isolated and characterized from the endophytic fungus *Penicillium* sp. QM-4. Their structures were elucidated through comprehensive spectroscopic analyses. Compounds **1**–**5** were evaluated for antioxidant activity, and compound **3** displayed a significant antioxidant effect in the DPPH assay. Based on the DPPH assay, antioxidant activity is strongly influenced by the number and position of phenolic hydroxyl groups.

## Figures and Tables

**Table 1:** NMR data (400 MHz for ^1^H and 100 MHz for ^13^C, in CD_3_OD) for **1** and **2**.

No.	1		2	
	*δ*c, Type	*δ*_H_, m (*J* in Hz)	*δ*c, Type	*δ*_H_, m (*J* in Hz)
1	168.6, C		167.9, C	
2	128.4, C		127.8, C	
3	129.2, CH	7.99, d (7.7)	129.3, CH	8.02, d (7.7)
4	127.8, CH	7.48, dd (7.7, 7.6)	127.9, CH	7.48, dd (7.7, 7.6)
5	131.7, CH	7.59, dd (7.7, 7.6)	131.9, CH	7.60, dd (7.7, 7.6)
6	125.1, CH	7.19, d (7.6)	125.2, CH	7.21, d (7.7)
7	145.7, C		145.8, C	
8	202.4, C		202.5, C	
9	108.2, C		109.0, C	
10	161.9, C		162.2, C	
11	107.5, CH	6.16, s	104.3, CH	6.33, s
12	148.6, C		151.8, C	
13	107.5, CH	6.16, s	104.3, CH	6.33, s
14	161.9, C		162.2, C	
15	20.7, CH_3_	2.21, s, 3H	63.1, CH_2_	4.50, s, 2H

**Table 2:** NMR data (400 MHz for ^1^H and 100MHz for ^13^C, in CD_3_OD) for **4**.

No.	4	
	*δ*c, Type	*δ*_H_, m (*J* in Hz)
1	146.7, C	
2	105.7, C	
3	163.6, C	
4	99.2, CH	6.44, d, (2.6)
5	161.9, C	
6	110.0, CH	6.18, d, (2.6)
7	195.7, CH	10.45, s
8	134.0, C	
9	115.2, CH	6.59, s
10	141.9, C	
11	143.6, C	
12	115.9, CH	6.49, s
13	125.9, C	
14	17.9, CH_3_	1.92, s, 3H
15	54.5, CH_3_	3.82, s, 3H

**Table 3:** ABTS and DPPH radical scavenging activities of **1**–**5**.

Samples	IC_50_ (mg/mL)	
	DPPH	ABTS
Vc	0.018 ± 0.002	0.013 ± 0.001
1	0.915 ± 0.038	0.621 ± 0.024
2	1.052 ± 0.079	0.916 ± 0.082
3	<0.010	0.312 ± 0.064
4	0.270 ± 0.030	1.766 ± 0.400
5	0.372 ± 0.085	0.416 ± 0.073

## Data Availability

All the data in this research were presented in the manuscript and Supplementary Materials.

## Supplementary Materials

The following supporting information can be downloaded at: https://www.mdpi.com/article/10.3390/molecules31152720/s1, **Figure S1.** ^1^H NMR spectrum (400 MHz) of **1** in CD_3_OD; **Figure S2.** ^13^C NMR spectrum (100 MHz) of **1** in CD_3_OD; **Figure S3.** DEPT-135° spectrum (100 MHz) of **1** in CD_3_OD; **Figure S4.** HSQC spectrum of **1** in CD_3_OD; **Figure S5.** HMBC spectrum of **1** in CD_3_OD; **Figure S6.** ^1^H-^1^H COSY spectrum of **1** in CD_3_OD; **Figure S7.** ROESY spectrum of **1** in CD_3_OD; **Figure S8.** HR-ESI-MS spectrum of **1**; **Figure S9.** UV spectrum of **1**; **Figure S10.** IR spectrum of **1;** **Figure S11.** ^1^H NMR spectrum (400 MHz) of **2** in CD_3_OD; **Figure S12.** ^13^C NMR spectrum (100 MHz) of **2** in CD_3_OD; **Figure S13.** DEPT-135° spectrum (100 MHz) of **2** in CD_3_OD; **Figure S14.** HSQC spectrum of **2** in CD_3_OD; **Figure S15.** HMBC spectrum of **2** in CD_3_OD; **Figure S16.** ^1^H-^1^H COSY spectrum of **2** in CD_3_OD; **Figure S17.** ROESY spectrum of **2** in CD_3_OD; **Figure S18.** HR-ESI-MS spectrum of **2**; **Figure S19.** UV spectrum of **2**; **Figure S20.** IR spectrum of **2**; **Figure S21.** ^1^H NMR spectrum (400 MHz) of **3** in CD_3_OD; **Figure S22.** ^13^C NMR spectrum (100 MHz) of **3** in CD_3_OD; **Figure S23.** ^1^H NMR spectrum (400 MHz) of **4** in CD_3_OD; **Figure S24.** ^13^C NMR spectrum (100 MHz) of **4** in CD_3_OD; **Figure S25.** DEPT-135° spectrum (100 MHz) of **4** in CD_3_OD; **Figure S26.** HSQC spectrum of **4** in CD_3_OD; **Figure S27.** HMBC spectrum of **4** in CD_3_OD; **Figure S28.** ^1^H-^1^H COSY spectrum of **4** in CD_3_OD; **Figure S29.** ROESY spectrum of **4** in CD_3_OD; **Figure S30.** HR-ESI-MS spectrum of **4**; **Figure S31.** UV spectrum of **4**; **Figure S32.** IR spectrum of **4**; **Figure S33.** Optical rotation spectrum of **4**; **Figure S34.** ^1^H NMR spectrum (400 MHz) of **5** in CD_3_OD; **Figure S35.** ^13^C NMR spectrum (100 MHz) of **5** in CD_3_OD; **Figure S36.** Gene sequences of *Penicillium* sp. QM-4; **Figure S37.** Phylogenetic tree of *Penicillium* sp. QM-4.
