# Supplementary material for: Antioxidant Benzophenones and Biphenyls from an Endophytic Fungus Penicillium sp. QM-4
Source: Molecules. 2026 Aug 5;31(15):2720. doi: 10.3390/molecules31152720 (PMC13468654; doi:10.3390/molecules31152720)
Supplement: Supplementary file 1 [file molecules-31-02720-s001.zip › molecules-4417500-supplementary.pdf]

Antioxidant benzophenones and biphenyls from an endophytic fungus *Penicillium* sp. QM-4

Chuan-Mao Zhang, Xiang-Gui Lei, Jun Ma, Jin-Feng Cao, Yang Xiao, Jian-Hai Ding*

Key Laboratory of Green Catalytic Materials and Technology of Ningxia Province, College of Chemistry and Chemical Engineering, Ningxia Normal University, Guyuan 756000, China

* Corresponding author.
E-mail address: dingjh_nx@126.com (J.-H.D)

Figure S1. ^{1}H NMR spectrum (400 MHz) of **1** in CD_3OD 4

Figure S2. ^{13}C NMR spectrum (100 MHz) of **1** in CD_3OD 4

Figure S3. DEPT-135° spectrum (100 MHz) of **1** in CD_3OD 5

Figure S4. HSQC spectrum of **1** in CD_3OD 5

Figure S5. HMBC spectrum of **1** in CD_3OD 6

Figure S6. ^{1}H-^{1}H COSY spectrum of **1** in CD_3OD 6

Figure S7. ROESY spectrum of **1** in CD_3OD 7

Figure S8. HR-ESI-MS spectrum of **1** 7

Figure S9. UV spectrum of **1** 8

Figure S10. IR spectrum of **1** 8

Figure S11. ^{1}H NMR spectrum (400 MHz) of **2** in CD_3OD 9

Figure S12. ^{13}C NMR spectrum (100 MHz) of **2** in CD_3OD 9

Figure S13. DEPT-135° spectrum (100 MHz) of **2** in CD_3OD 10

Figure S14. HSQC spectrum of **2** in CD_3OD 10

Figure S15. HMBC spectrum of **2** in CD_3OD 11

Figure S16. ^{1}H-^{1}H COSY spectrum of **2** in CD_3OD 11

Figure S17. ROESY spectrum of **2** in CD_3OD 12

Figure S18. HR-ESI-MS spectrum of **2** 12

Figure S19. UV spectrum of **2** 13

Figure S20. IR spectrum of **2** 13

Figure S21. ^{1}H NMR spectrum (400 MHz) of **3** in CD_3OD 14

Figure S22. ^{13}C NMR spectrum (100 MHz) of **3** in CD_3OD 14

Figure S23. ^{1}H NMR spectrum (400 MHz) of **4** in CD_3OD 15

Figure S24. ^{13}C NMR spectrum (100 MHz) of **4** in CD_3OD 15

Figure S25. DEPT-135° spectrum (100 MHz) of **4** in CD_3OD 16

Figure S26. HSQC spectrum of **4** in CD_3OD 16

Figure S27. HMBC spectrum of **4** in CD_3OD 17

Figure S28. ^{1}H-^{1}H COSY spectrum of **4** in CD_3OD 17

Figure S29. ROESY spectrum of **4** in CD_3OD 18

Figure S30. HR-ESI-MS spectrum of **4** 18

Figure S31. UV spectrum of **4** 19

Figure S32. IR spectrum of **4** 19

Figure S33 Optical rotation spectrum of **4** 19

Figure S34. ^{1}H NMR spectrum (400 MHz) of **5** in CD_3OD 20

Figure S35. ^{13}C NMR spectrum (100 MHz) of **5** in CD_3OD 20

Figure S36. Gene sequences of *Penicillium* sp. QM-4 21

Figure S37. Phylogenetic tree of *Penicillium* sp. QM-4 21

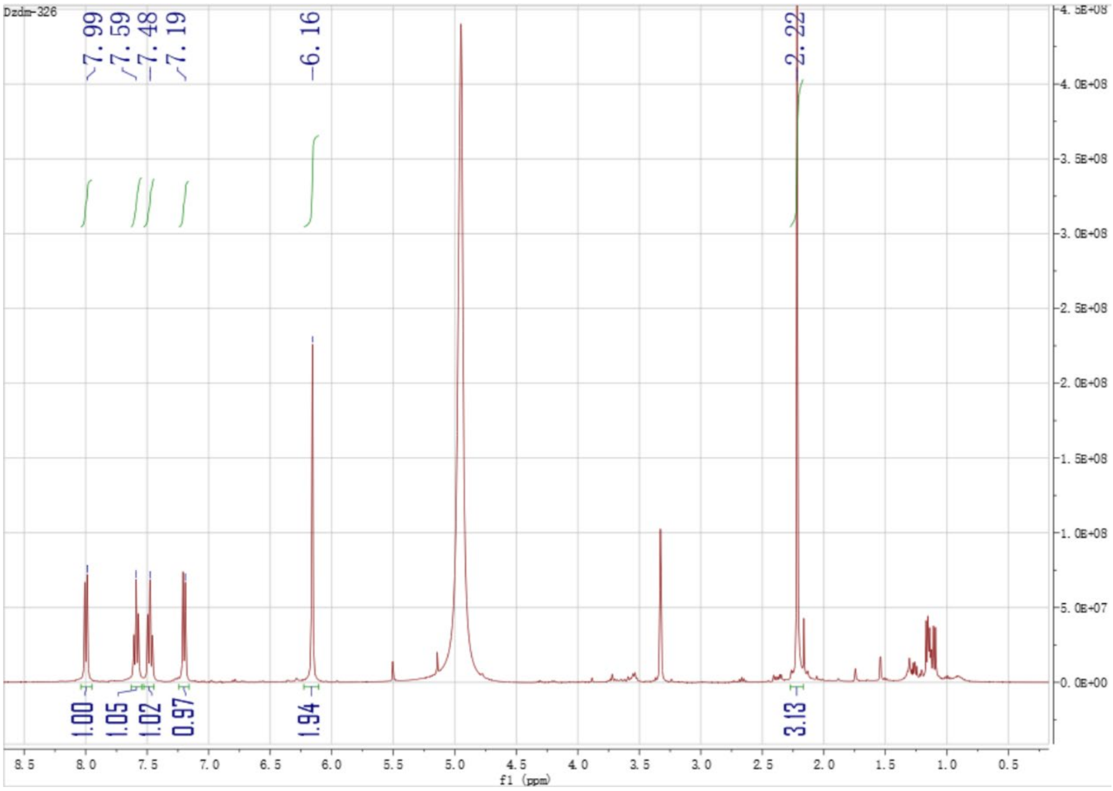

Figure S1. ¹H NMR spectrum (400 MHz) of **1** in CD_3OD

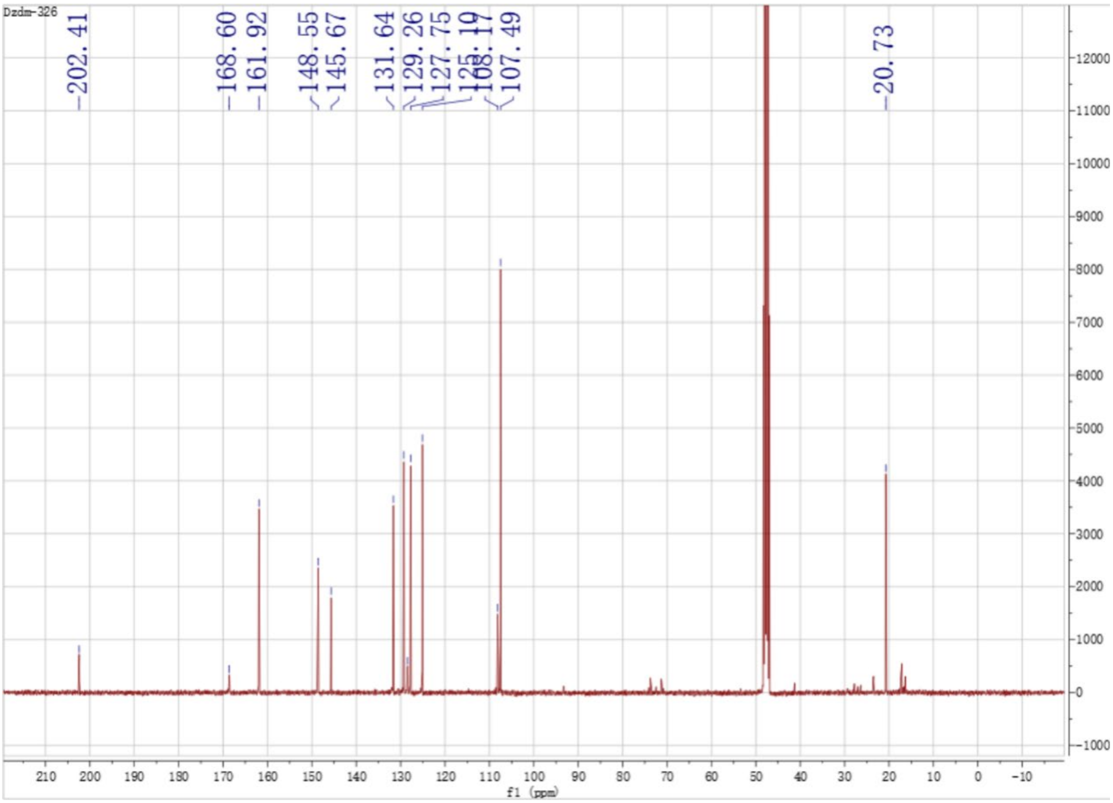

Figure S2.¹³C NMR spectrum (100 MHz) of **1** in CD_3OD

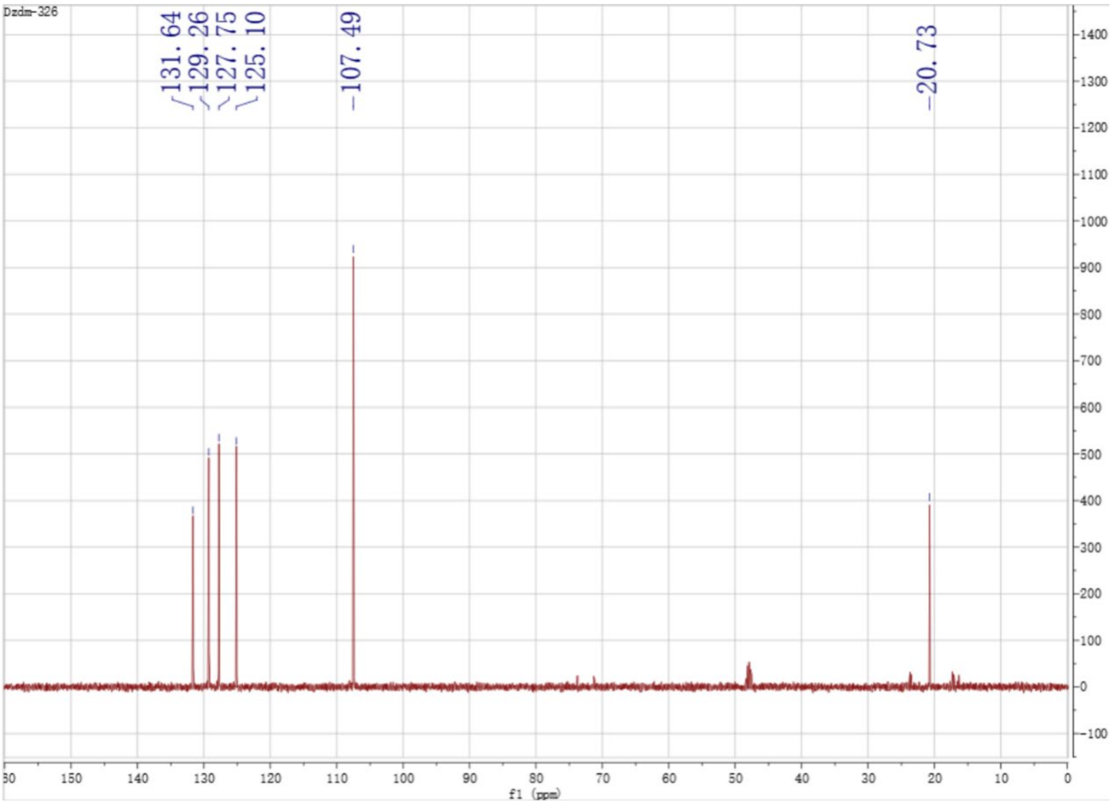

Figure S3. DEPT-135° spectrum (100 MHz) of **1** in CD_3OD

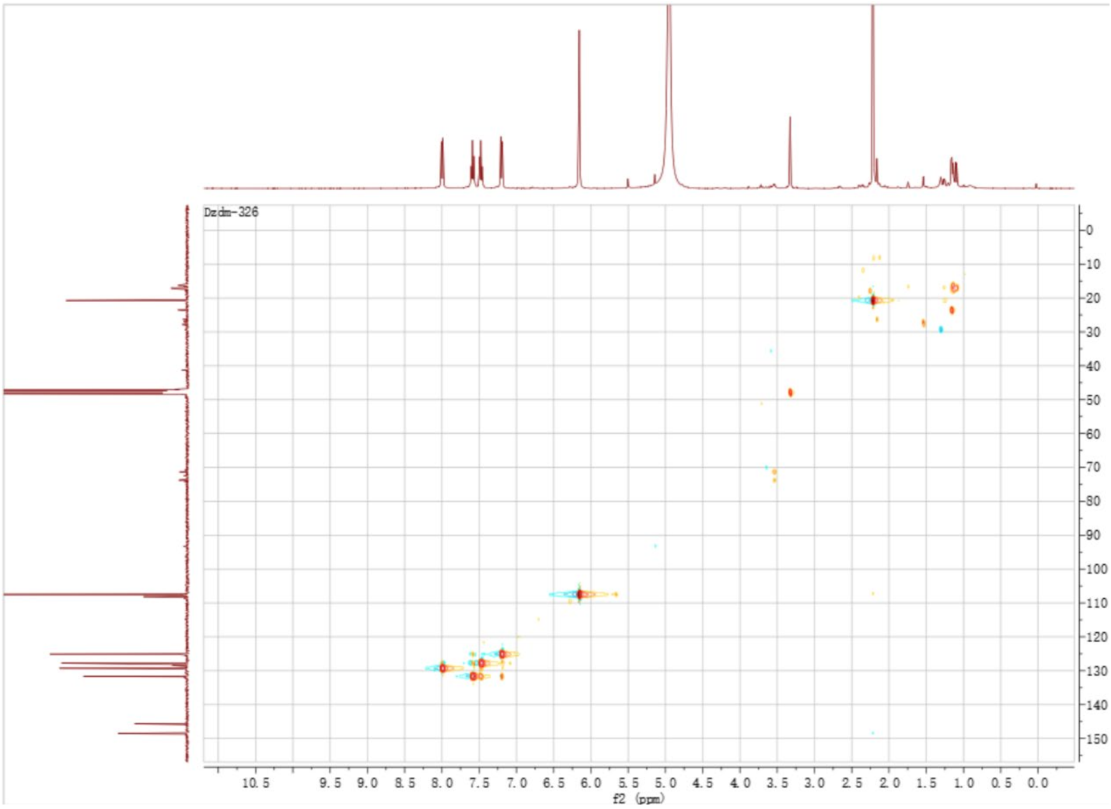

Figure S4. HSQC spectrum of **1** in CD_3OD

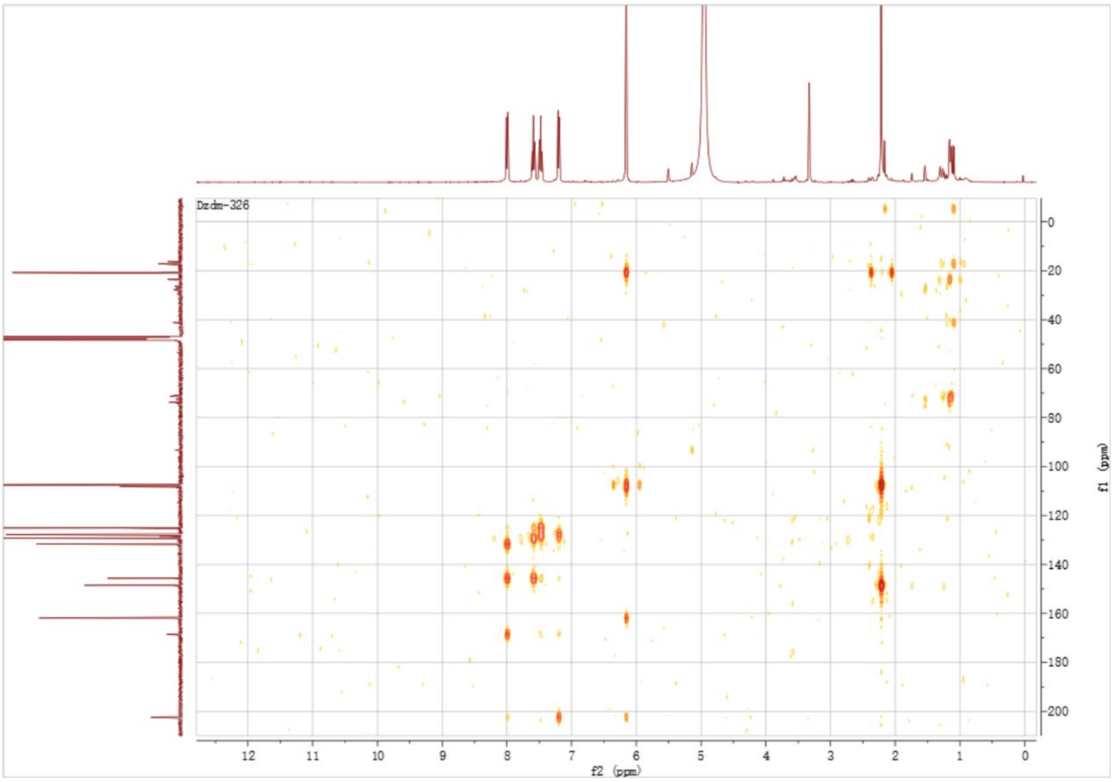

Figure S5. HMBC spectrum of **1** in CD_3OD

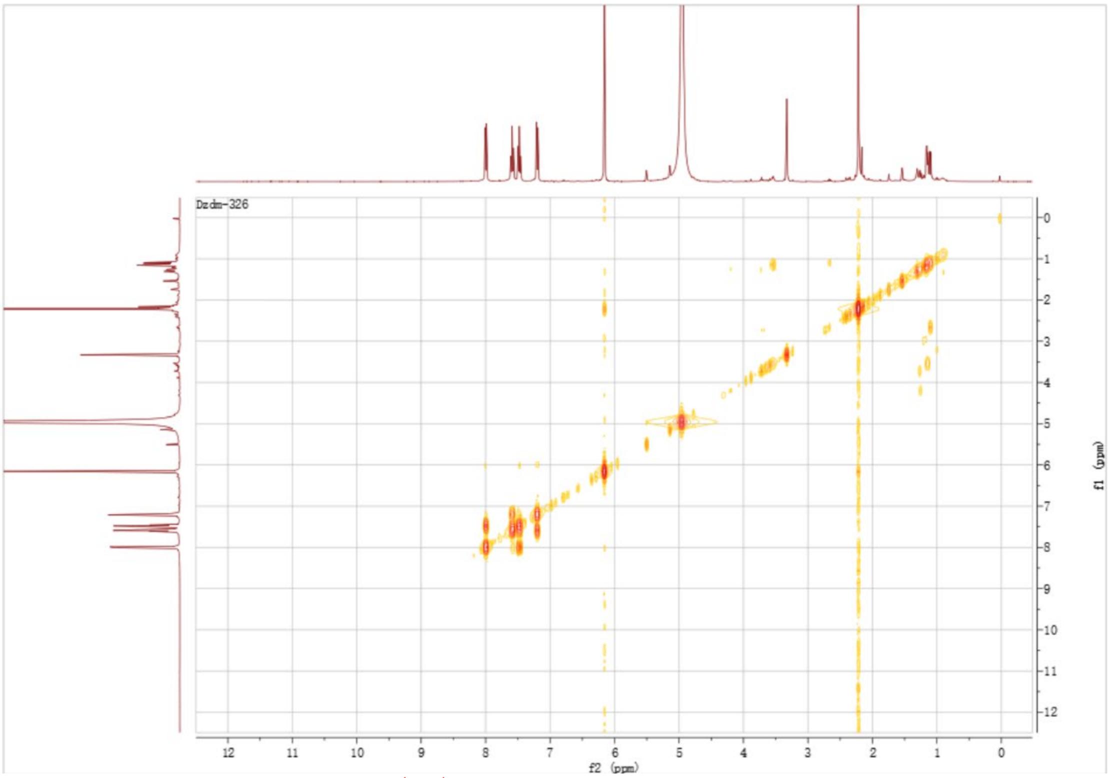

Figure S6. ^1H-^1H COSY spectrum of **1** in CD_3OD

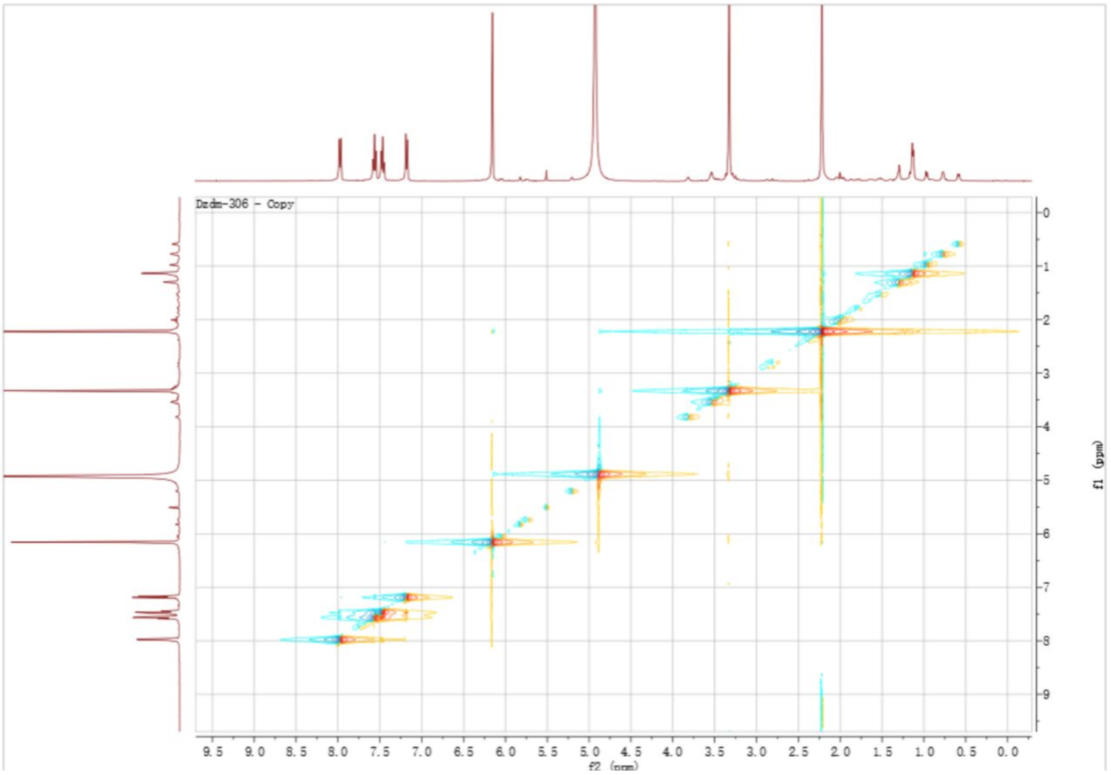

Figure S7. ROESY spectrum of **1** in CD_3OD

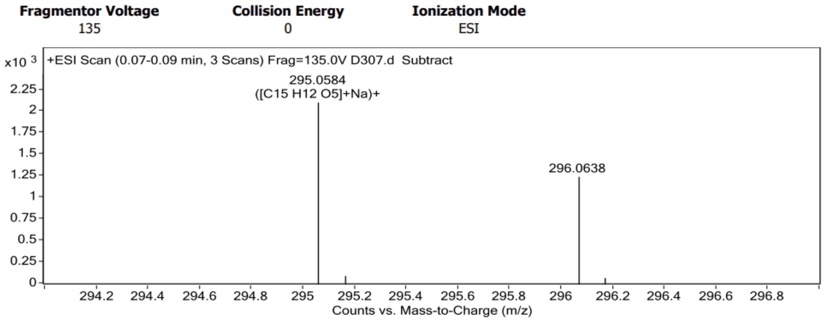

Peak List

m/z	z	Abund	Formula	Ion
125.0607	1	4091.47		
126.0662	1	1690.41		
145.1348	1	2326.23		
149.0244	1	7758.31		
166.059	1	4831.55		
184.0696	1	912.45		
206.0513	1	3570.43		
255.0658	1	997.36		
295.0584	1	2099.04	C15 H12 O5	(M+Na)+
296.0638	1	1235.83		

Formula Calculator Element Limits

Element	Min	Max
C	3	50
H	0	100
O	0	20

Formula Calculator Results

Formula	CalculatedMass	CalculatedMz	Mz	Diff. (mDa)	Diff. (ppm)	DBE
C15 H12 O5	272.0685	295.0577	295.0584	-0.70	-2.37	10.0000

Figure S8. HR-ESI-MS spectrum of **1**

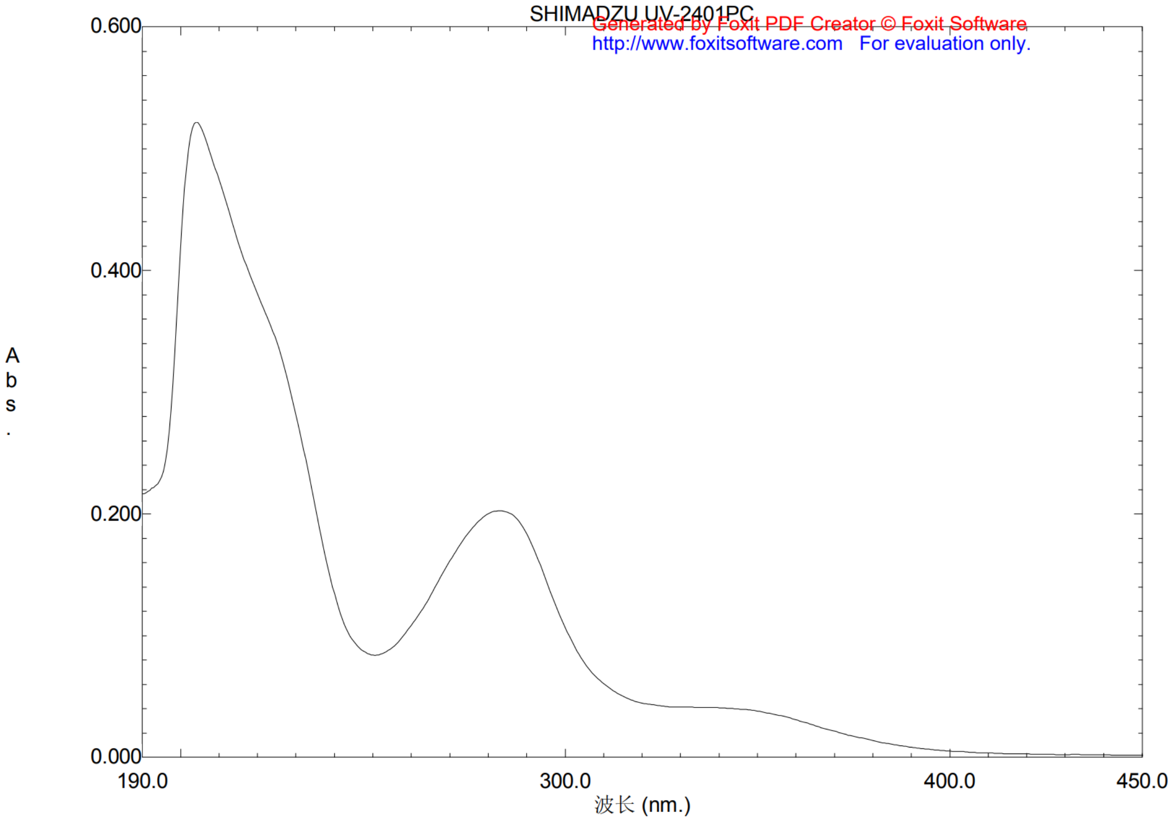

Generated by Foxit PDF Creator © Foxit Software
http://www.foxitsoftware.com For evaluation only.

Figure S9. UV spectrum of **1**

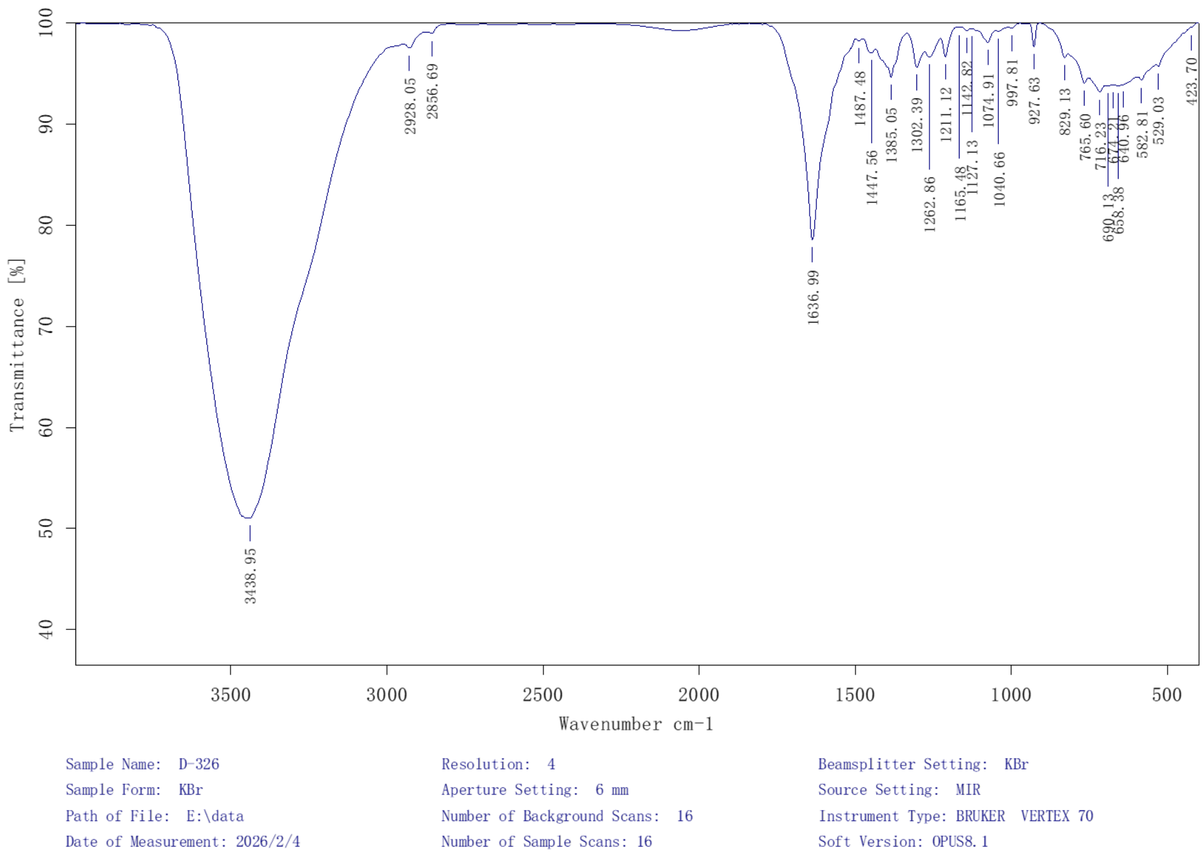

Figure S10. IR spectrum of **1**

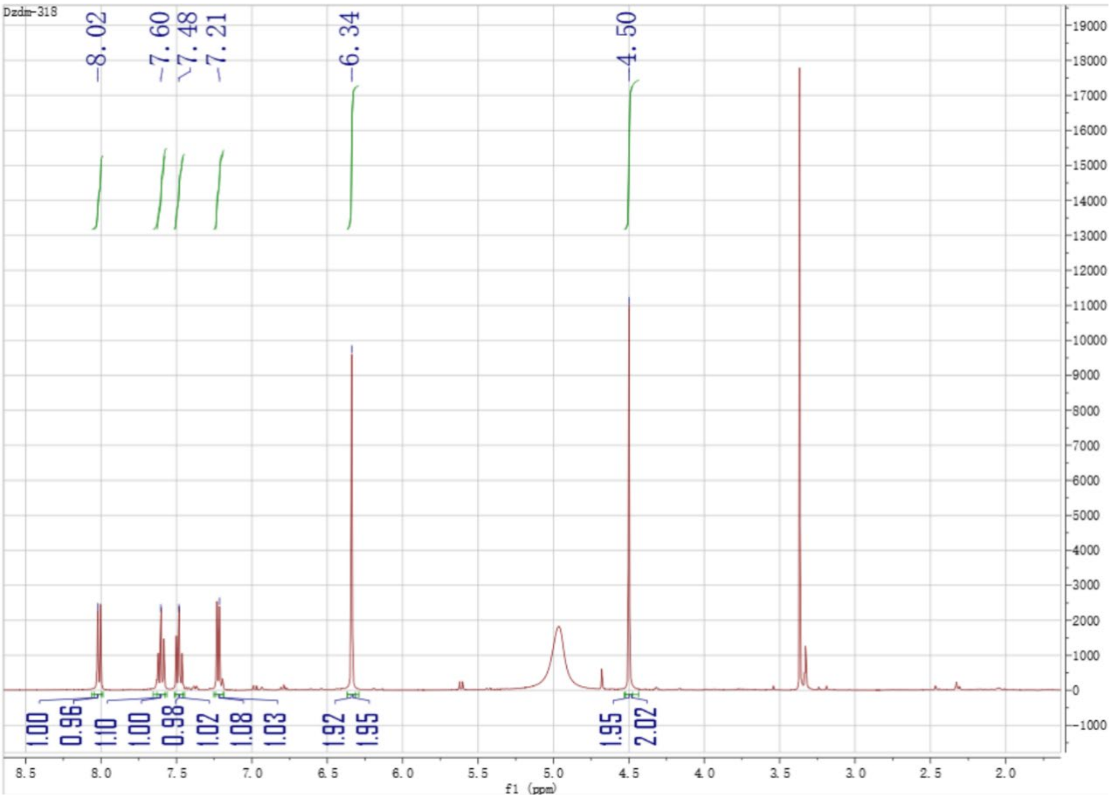

Figure S11. ^{1}H NMR spectrum (400 MHz) of **2** in CD_3OD

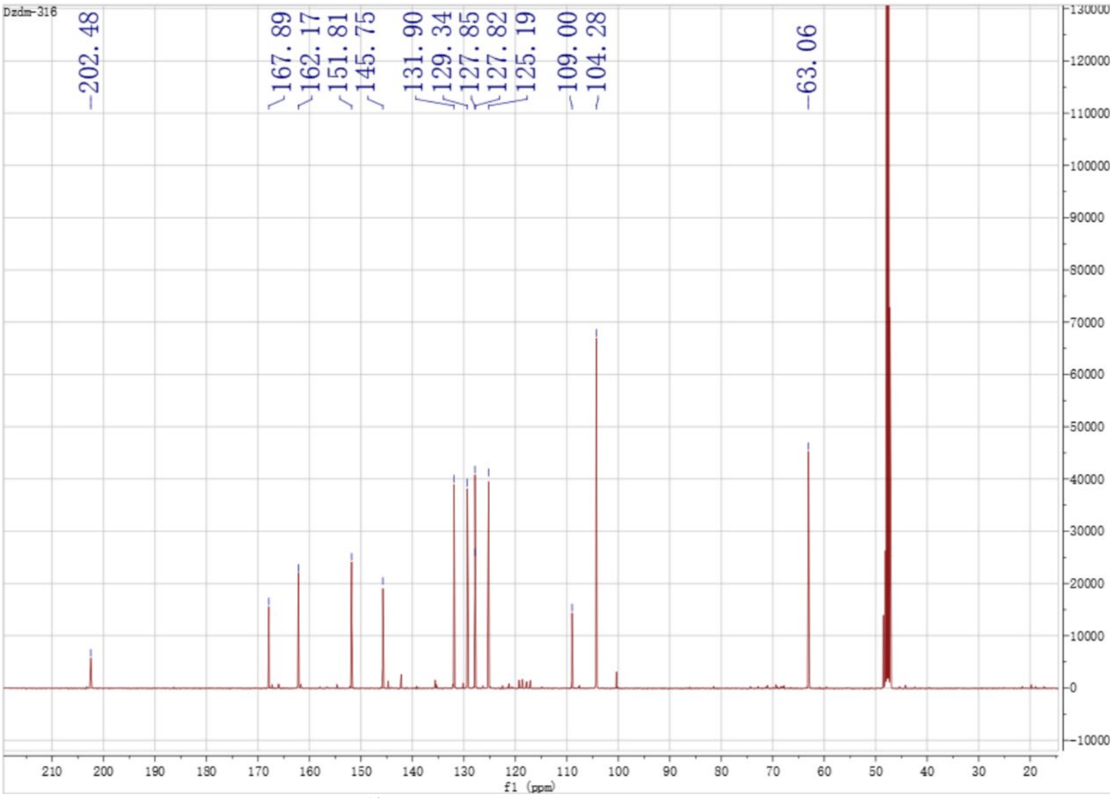

Figure S12. ^{13}C NMR spectrum (100 MHz) of **2** in CD_3OD

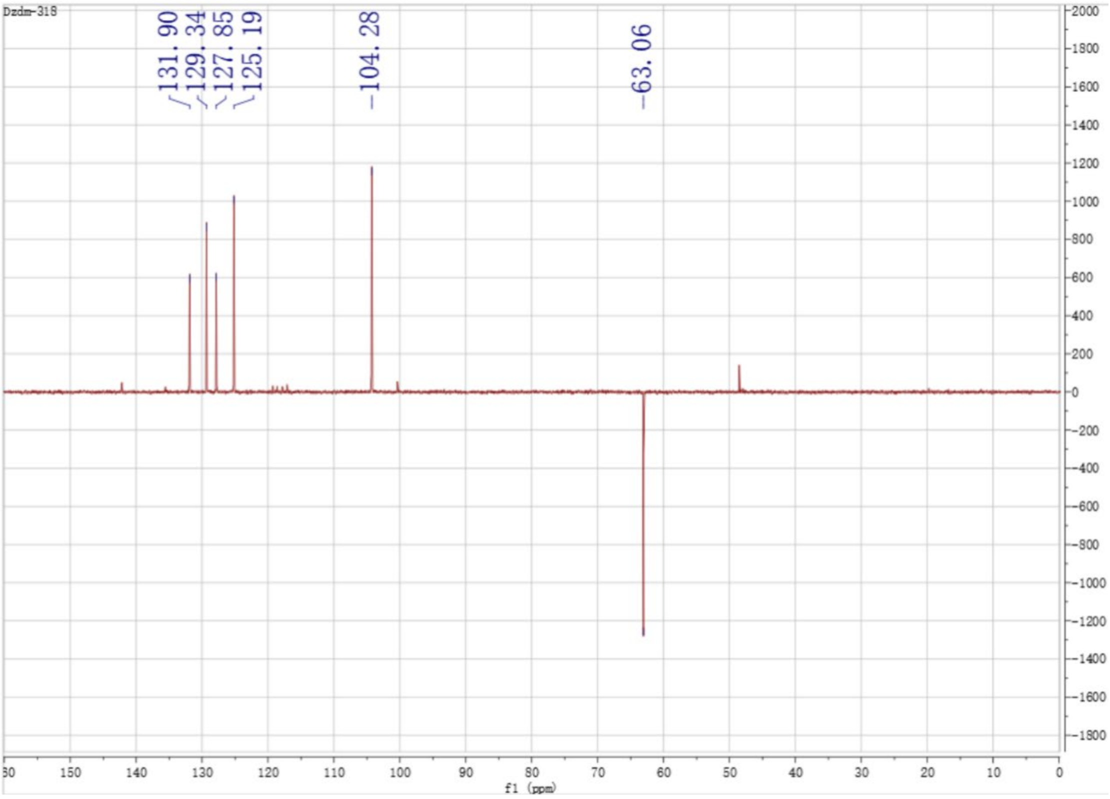

Figure S13. DEPT-135° spectrum (100 MHz) of **2** in CD_3OD

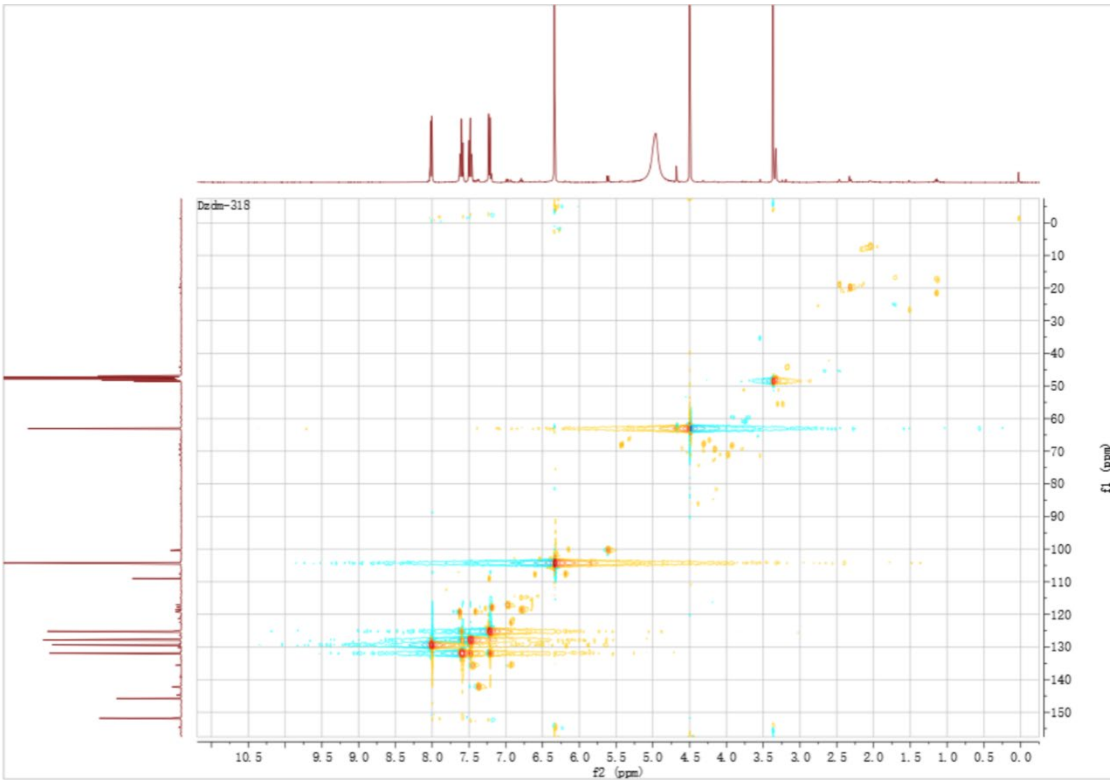

Figure S14. HSQC spectrum of **2** in CD_3OD

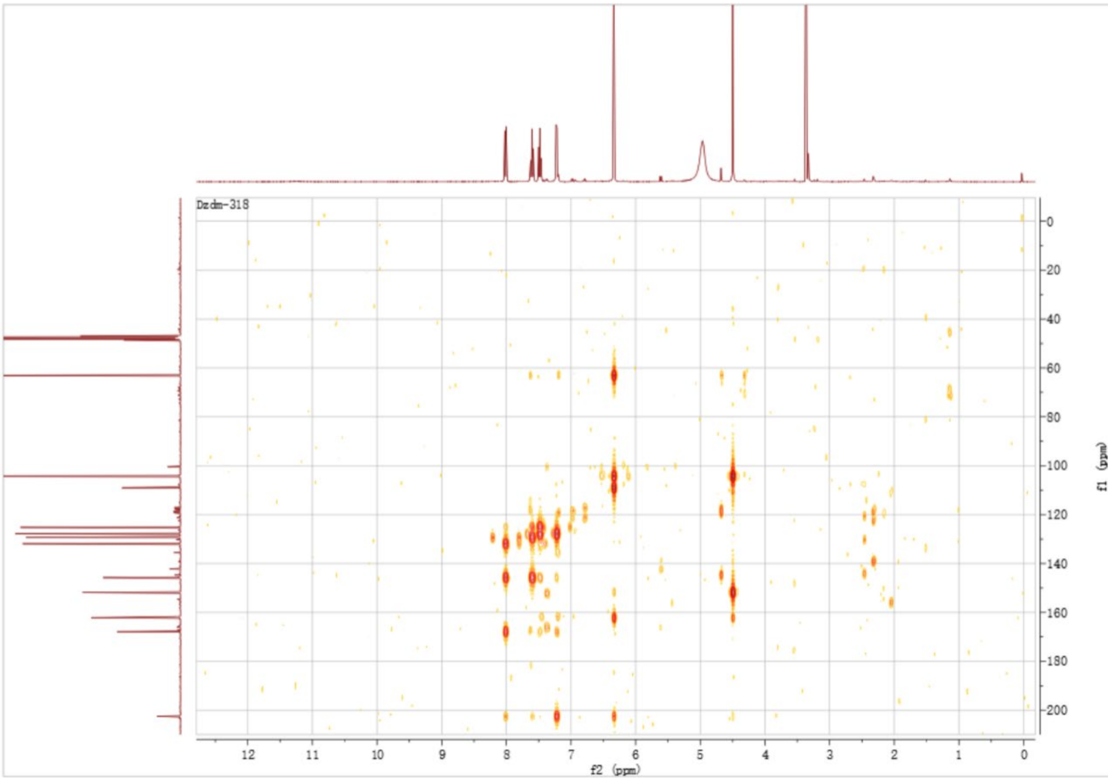

Figure S15. HMBC spectrum of **2** in CD_3OD

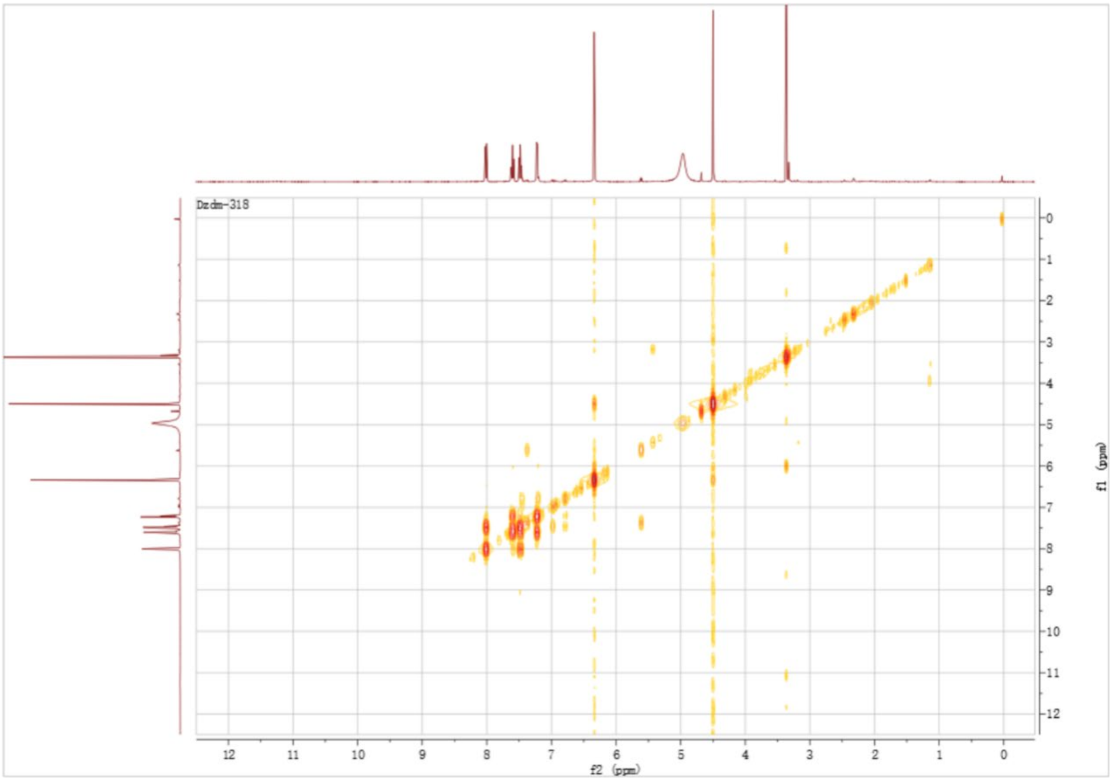

Figure S16. ^1H-^1H COSY spectrum of **2** in CD_3OD

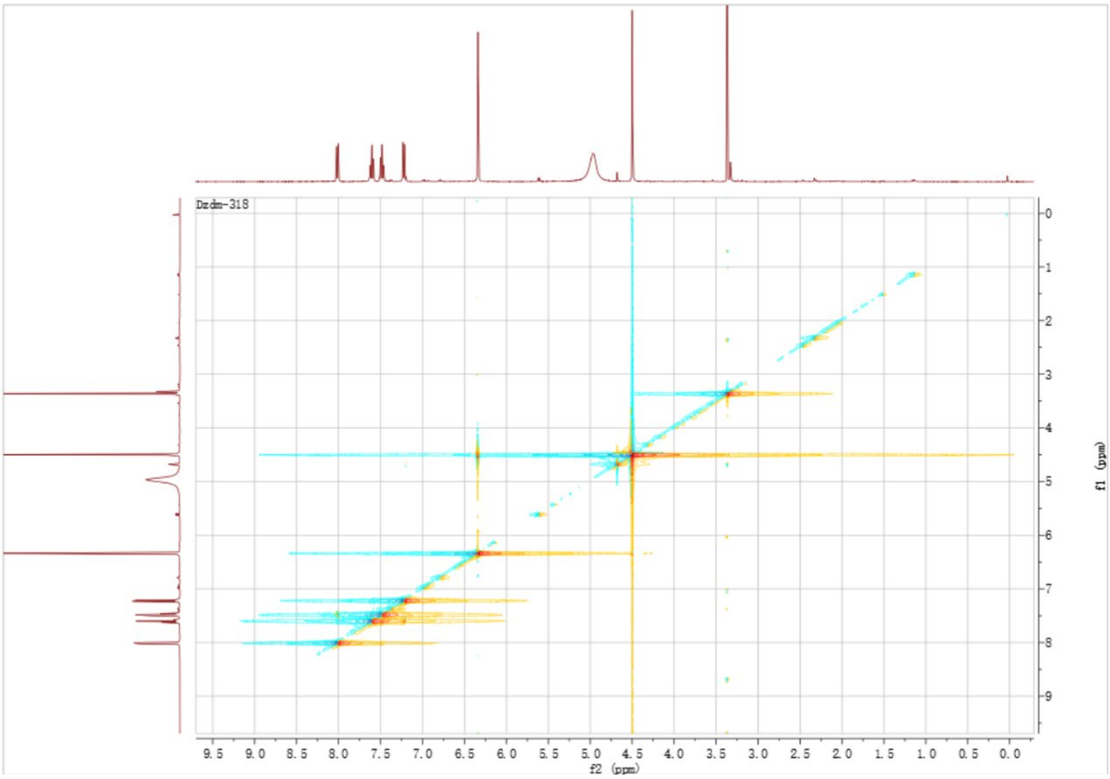

Figure S17. ROESY spectrum of **2** in CD_3OD

Fragmentor Voltage	Collision Energy	Ionization Mode
135	0	ESI

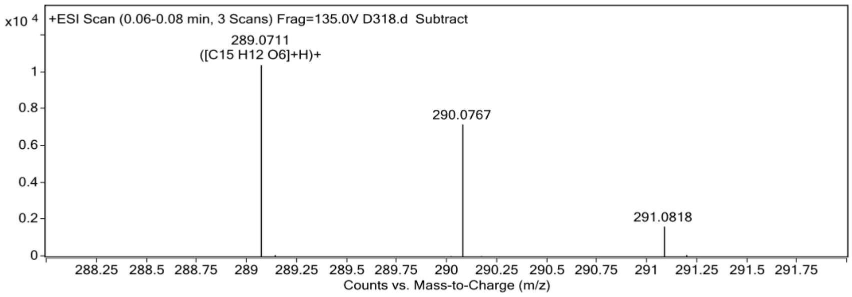

Peak List

m/z	z	Abund	Formula	Ion
123.0441	1	20064.99		
124.0504	1	12082.59		
149.0236	1	9132.4		
289.0711	1	10399.95	C15 H12 O6	(M+H)+
290.0767	1	7187.41		
311.0529	1	10014.84		
312.0589	1	6324.74		
599.1168	1	11521.52		
600.1226	1	15454.61		
601.1274	1	10189.73		

Formula Calculator Element Limits

Element	Min	Max
C	3	50
H	0	100
O	0	20

Formula Calculator Results

Formula	CalculatedMass	CalculatedMz	Mz	Diff. (mDa)	Diff. (ppm)	DBE
C15 H12 O6	288.0634	289.0707	289.0711	-0.40	-1.38	10.0000

Figure S18. HR-ESI-MS spectrum of **2**

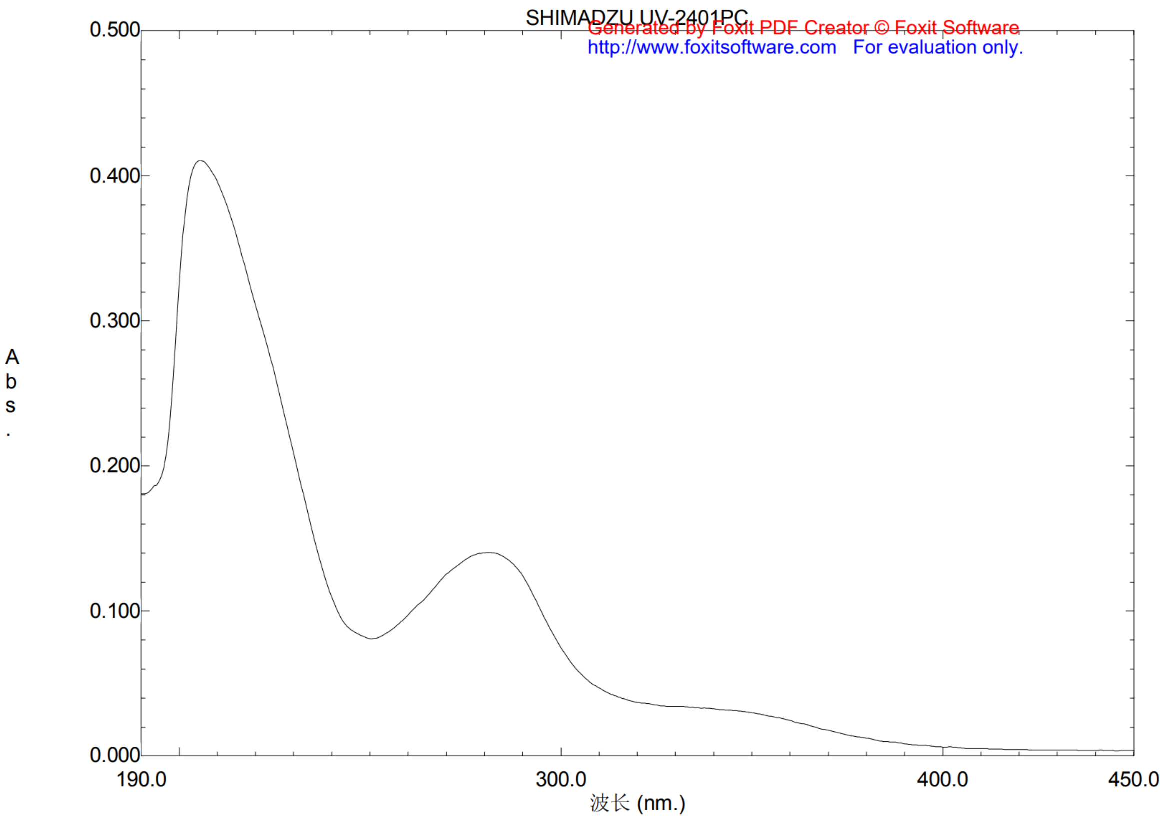

Generated by Foxit PDF Creator © Foxit Software
http://www.foxitsoftware.com For evaluation only.

Figure S19. UV spectrum of **2**

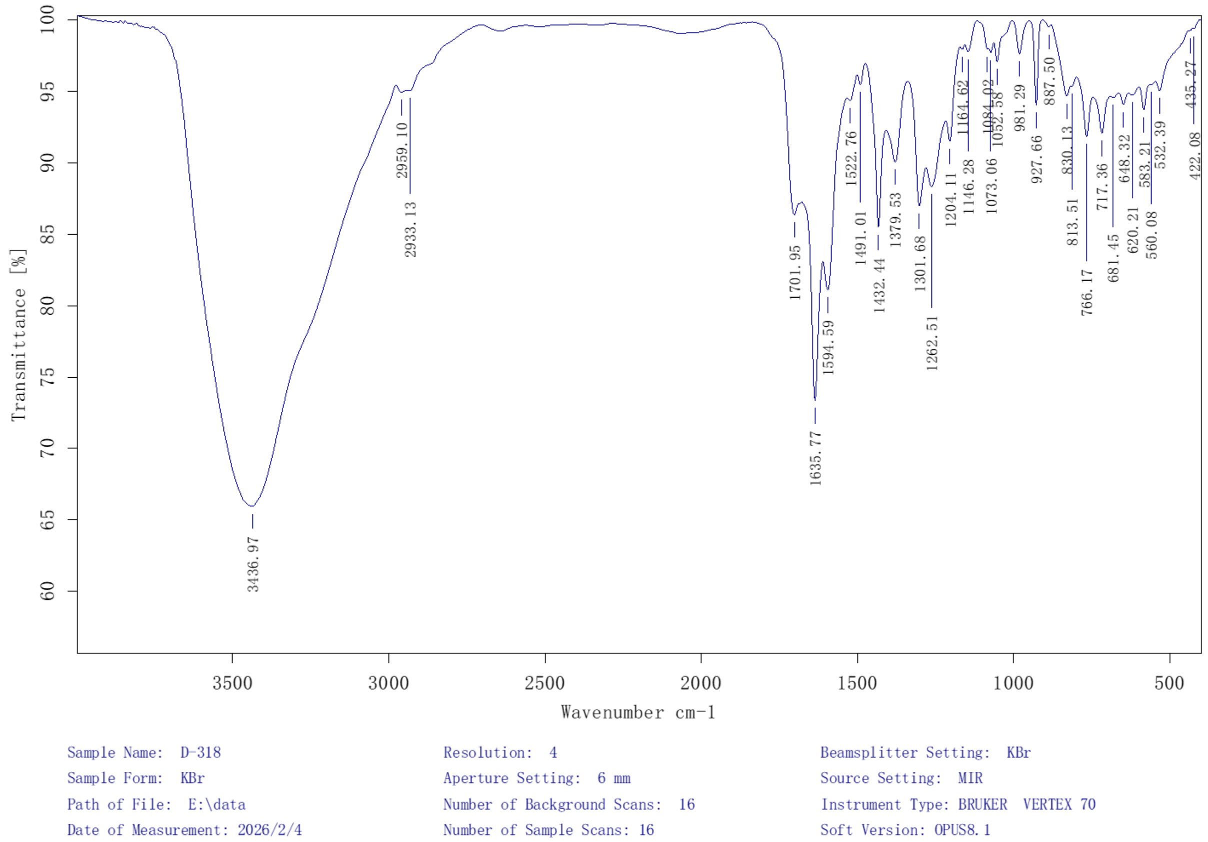

Figure S20. IR spectrum of **2**

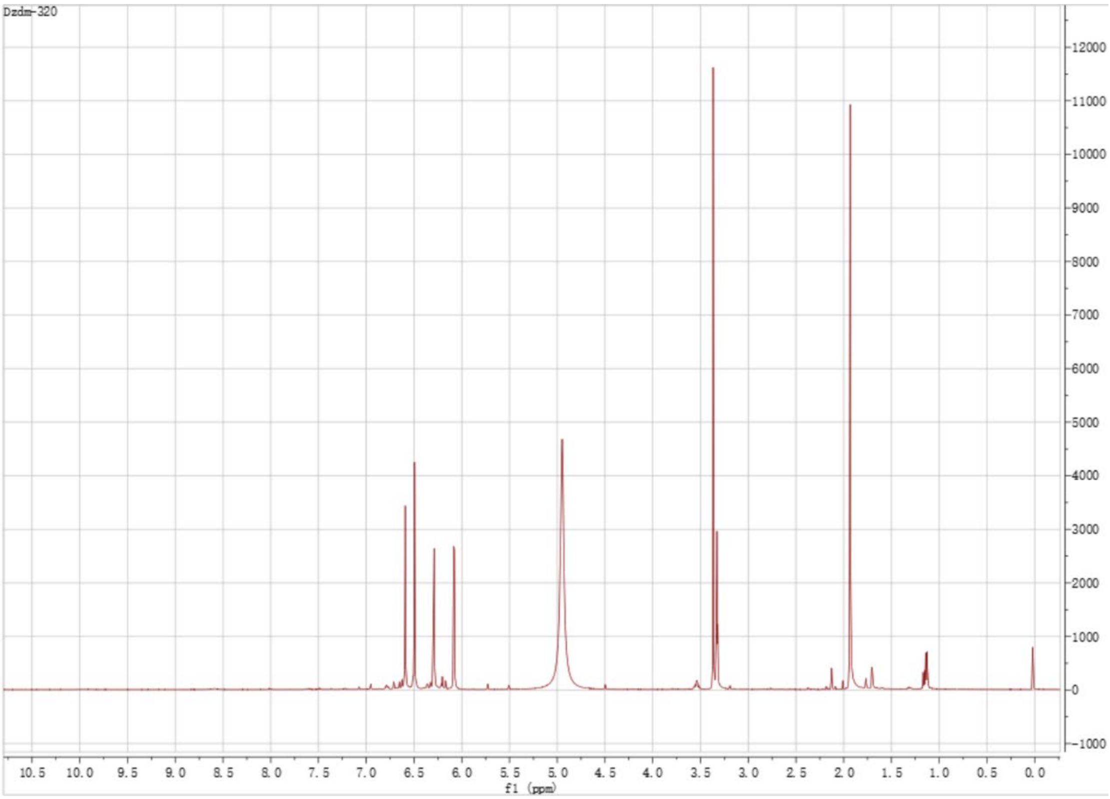

Figure S21. ^{1}H NMR spectrum (400 MHz) of **3** in CD_3OD

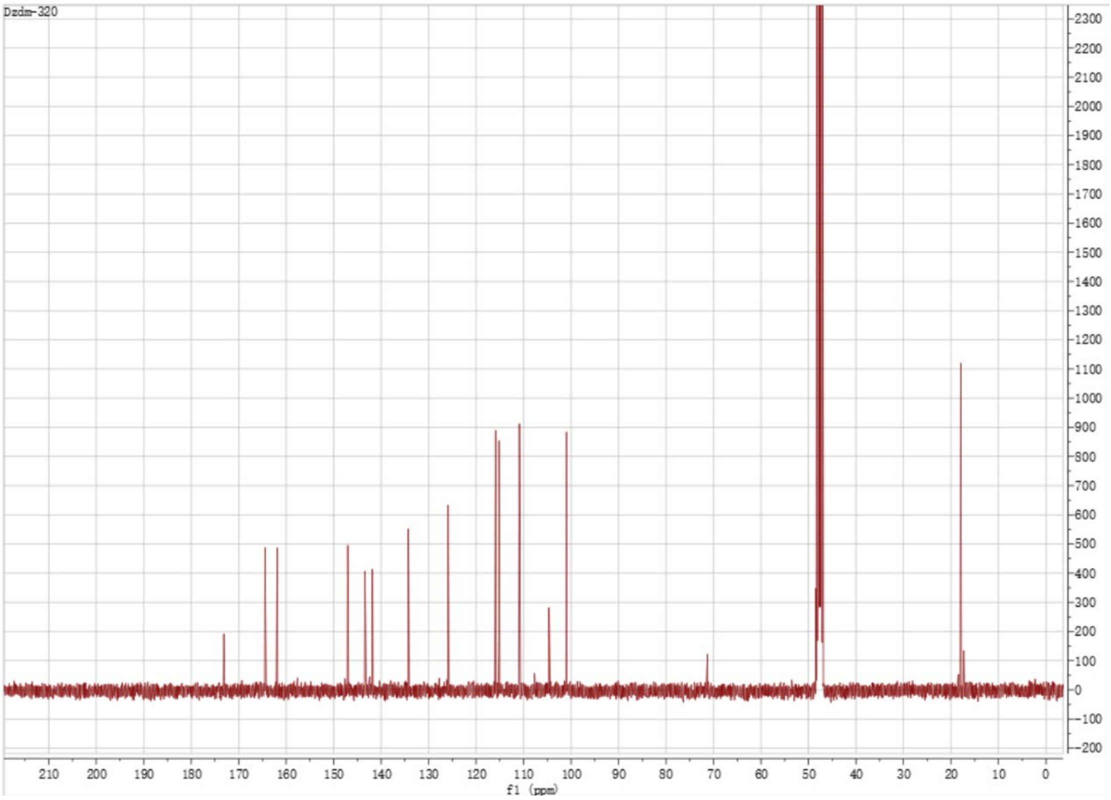

Figure S22. ^{13}C NMR spectrum (100 MHz) of **3** in CD_3OD

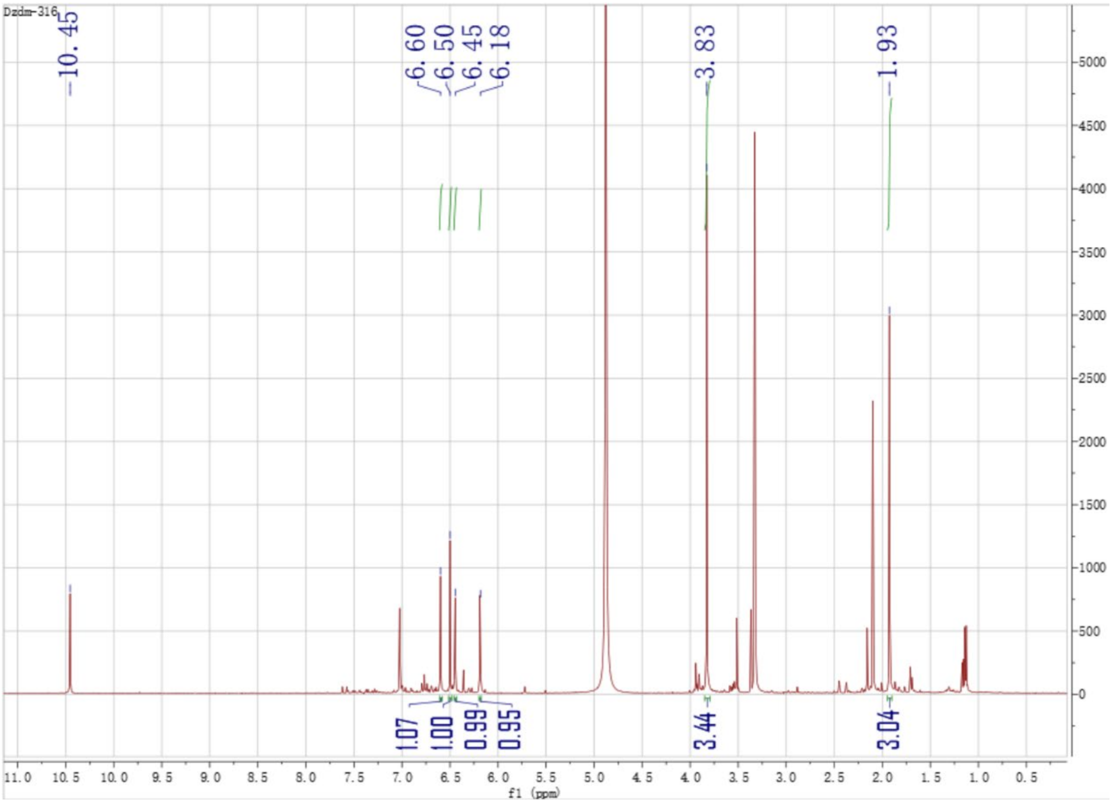

Figure S23. ¹H NMR spectrum (400 MHz) of **4** in CD_3OD

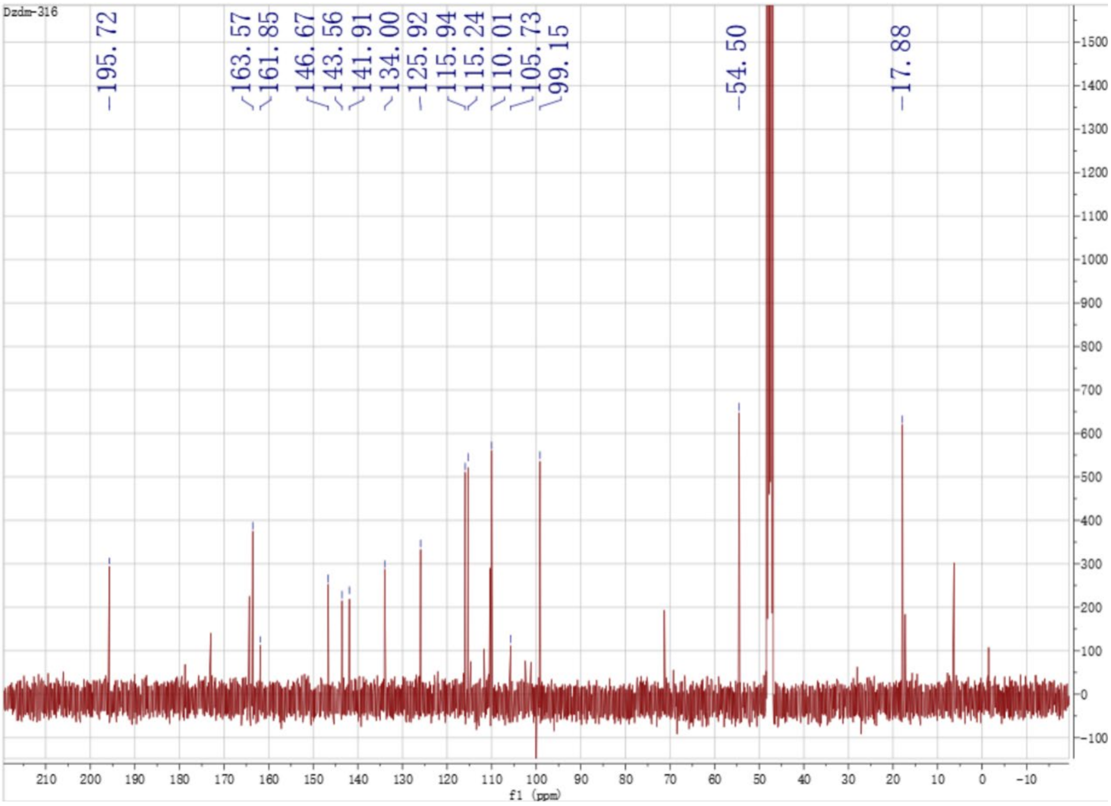

Figure S24.¹³C NMR spectrum (100 MHz) of **4** in CD_3OD

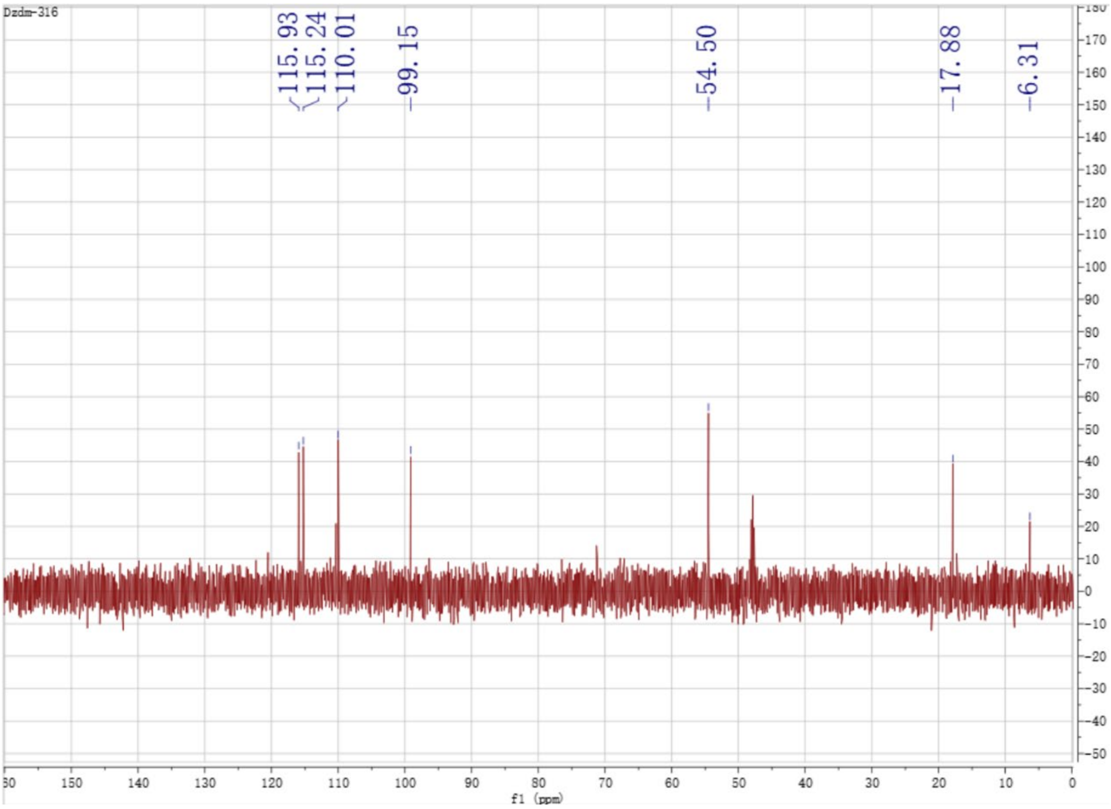

Figure S25. DEPT-135° spectrum (100 MHz) of **4** in CD_3OD

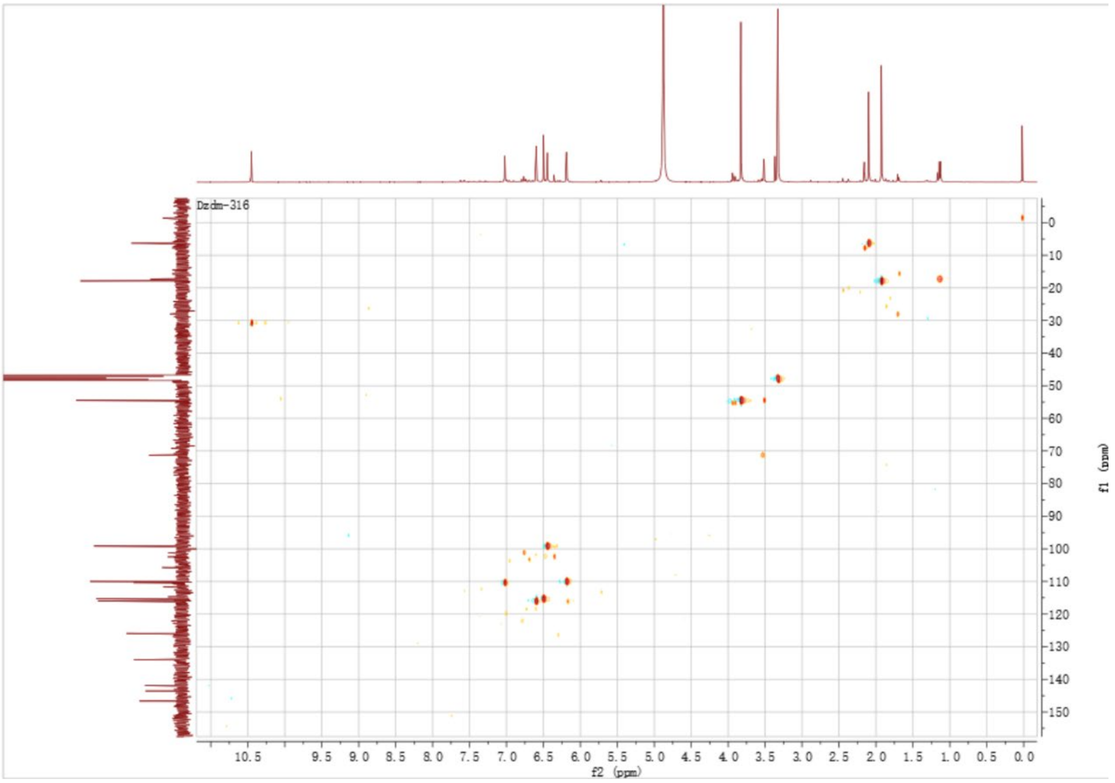

Figure S26. HSQC spectrum of **4** in CD_3OD

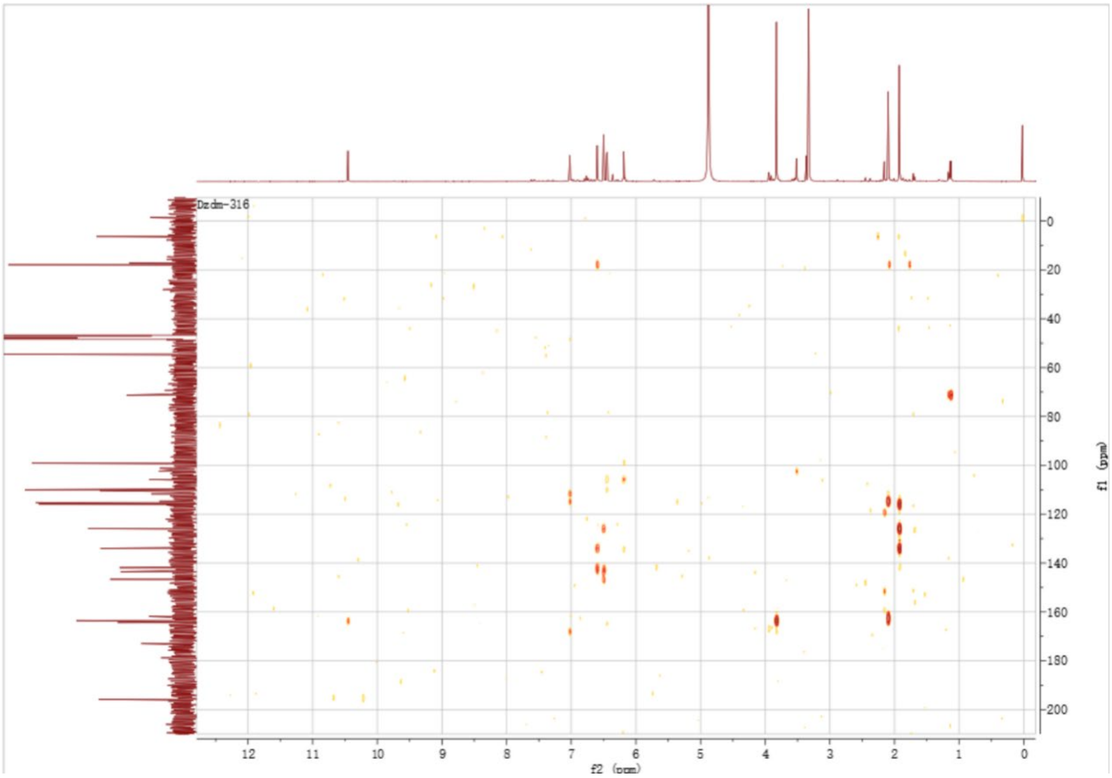

Figure S27. HMBC spectrum of **4** in CD_3OD

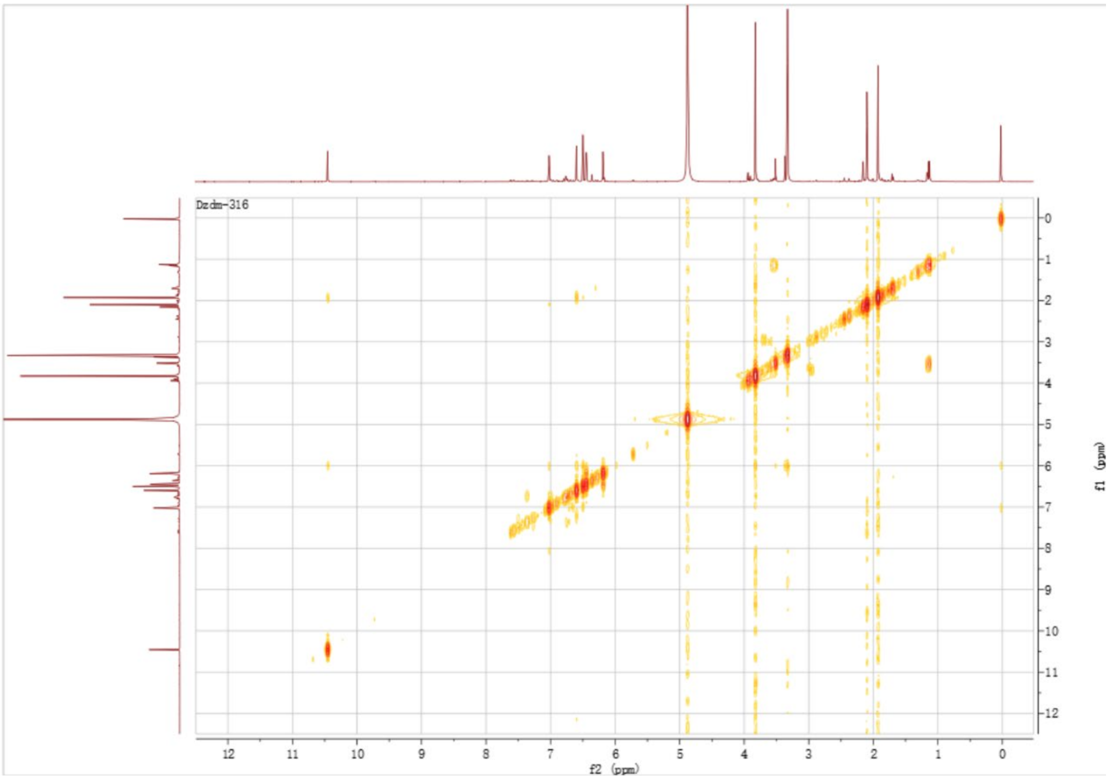

Figure S28. ^{1}H-^{1}H COSY spectrum of **4** in CD_3OD

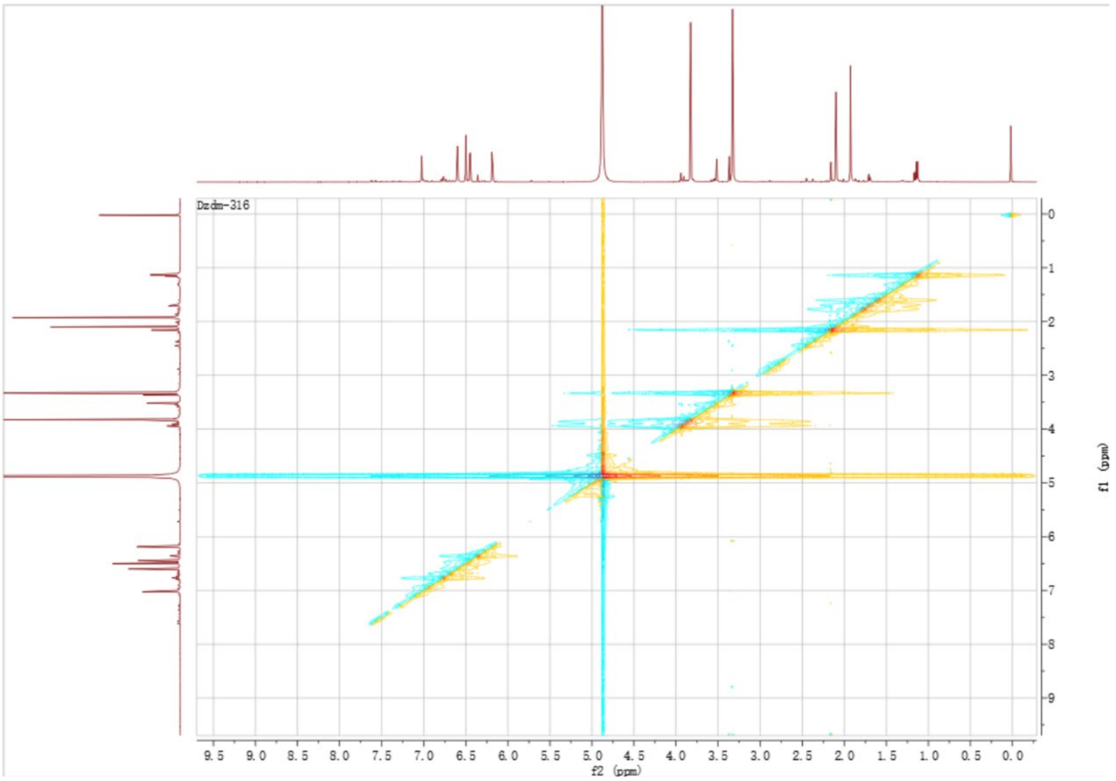

Figure S29. ROESY spectrum of **4** in CD_3OD

Fragmentor Voltage	Collision Energy	Ionization Mode
135	0	ESI

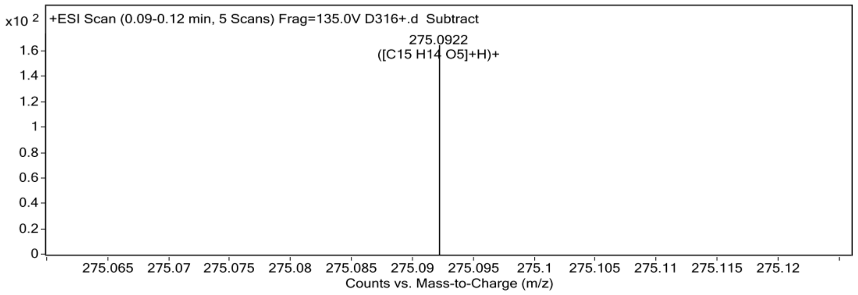

Peak List

m/z	z	Abund
145.1334	1	3666.75
179.034	1	9656.17
289.0708	1	3712.02
339.1022	1	3901.51
359.0738	1	4298.48
360.0768	1	7031.43
361.083	1	7839.39
364.1047	1	4159.96
662.3982	1	18743.5
663.4018	1	6723.76

Formula Calculator Element Limits

Element	Min	Max
C	3	50
H	0	100
O	0	20

Formula Calculator Results

Formula	CalculatedMass	CalculatedMz	Mz	Diff. (mDa)	Diff. (ppm)	DBE
C15 H14 O5	274.0841	275.0914	275.0922	-0.80	-2.91	9.0000

Figure S30. HR-ESI-MS spectrum of **4**

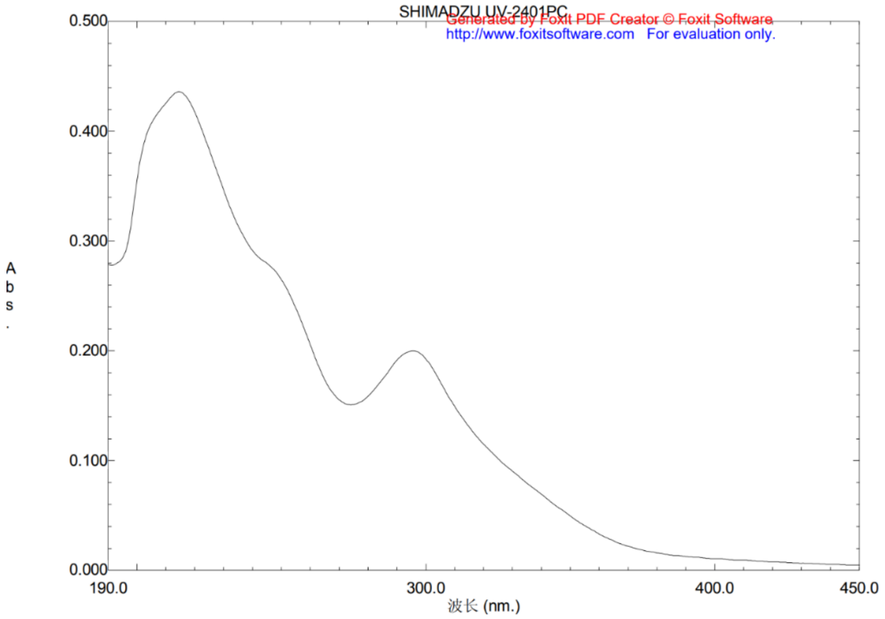

Figure S31. UV spectrum of **4**

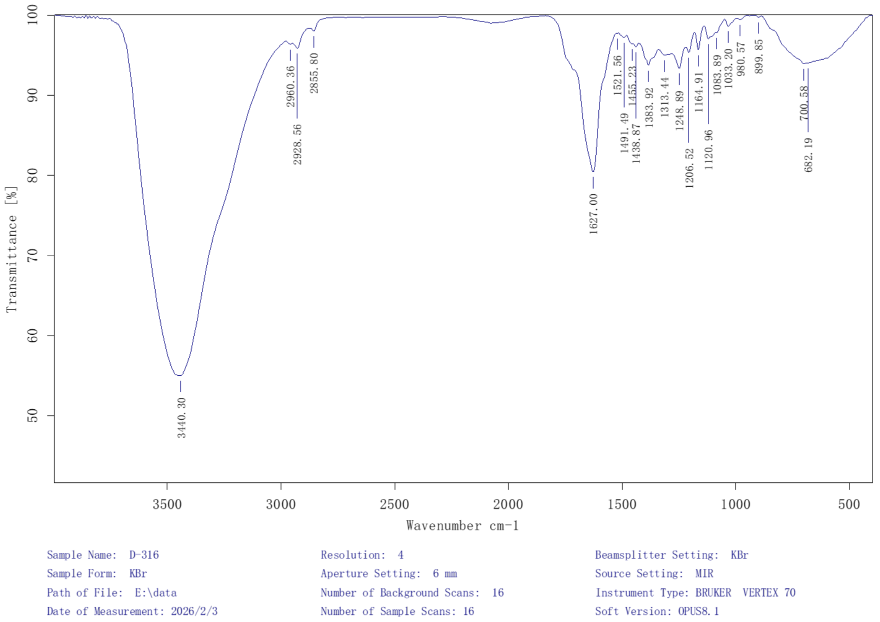

Figure S32. IR spectrum of **4**

Rudolph Research Analytical

This sample was measured on an Autopol VI, Serial #91058
Manufactured by Rudolph Research Analytical, Hackettstown, NJ, USA.

Measurement Date : Thursday, 23-APR-2026

Set Temperature : 20.0

Time Delay : Disabled

Delay between Measurement : Disabled

n	Average	Std.Dev.	% RSD	Maximum	Minimum
5	-5.50	1.12	-20.36	-5.00	-7.50

S.No	Sample ID	Time	Result	Scale	OR °Arc	WLG.nm	Lg.mm	Conc.g/100ml	Temp.
1	D-316	11:22:18 AM	-5.00	SR	-0.002	589	100.00	0.040	20.0
2	D-316	11:22:24 AM	-7.50	SR	-0.003	589	100.00	0.040	20.0
3	D-316	11:22:31 AM	-5.00	SR	-0.002	589	100.00	0.040	20.0
4	D-316	11:22:37 AM	-5.00	SR	-0.002	589	100.00	0.040	20.0
5	D-316	11:22:44 AM	-5.00	SR	-0.002	589	100.00	0.040	20.0

Figure S33. Optical rotation spectrum of **4**

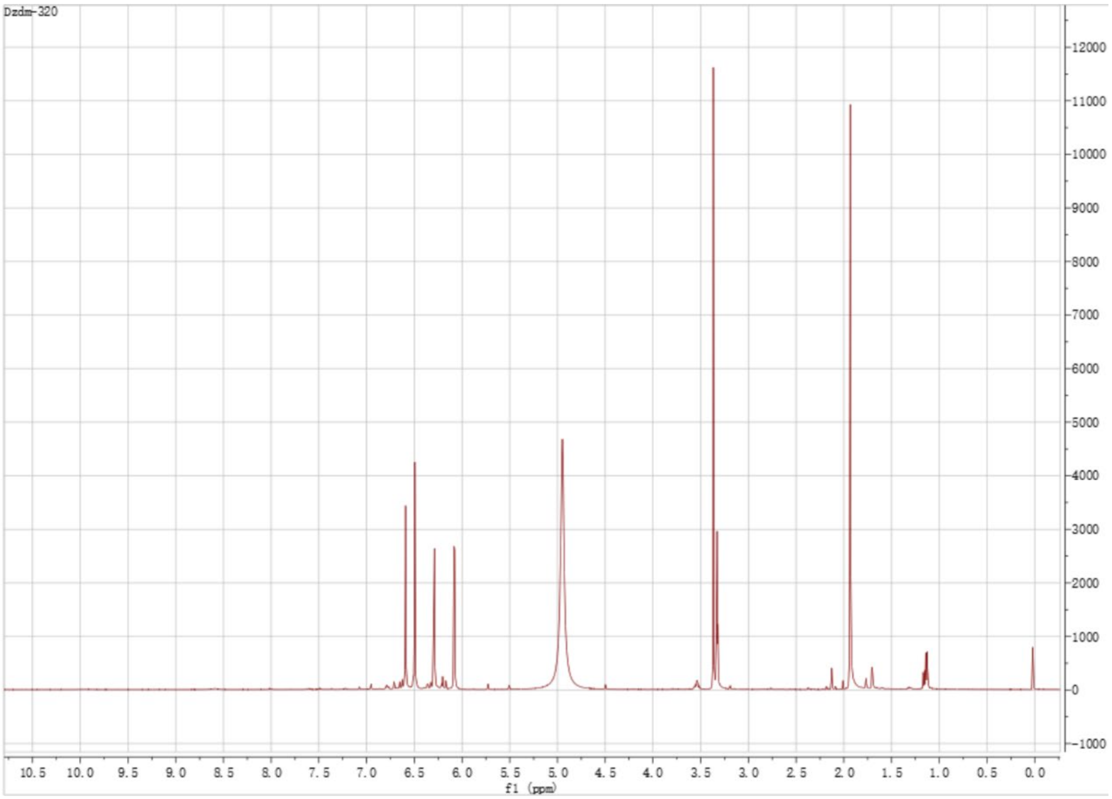

Figure S34. ^{1}H NMR spectrum (400 MHz) of **5** in CD_3OD

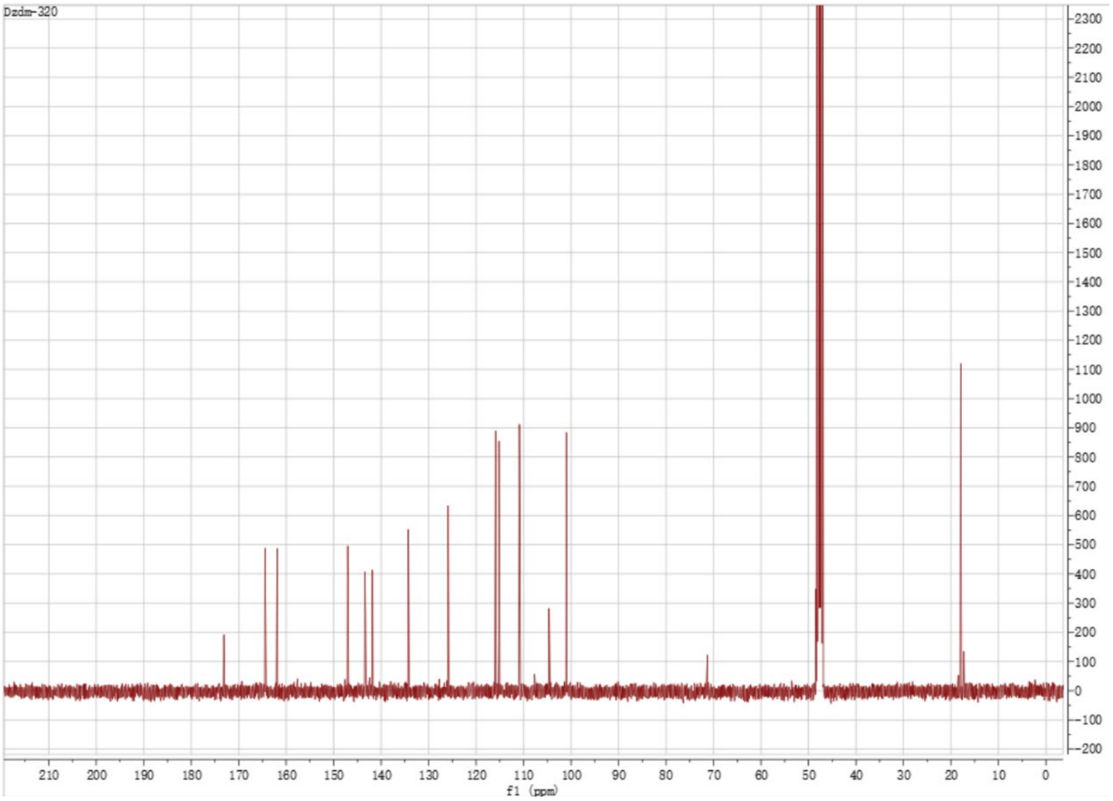

Figure S35. ^{13}C NMR spectrum (100 MHz) of **5** in CD_3OD

GTTTATTTTACCTTGTTGCTTCGGCGGGCCCGCCTTAACTGGCCGCCGGGGG
GCTTACGCCCCCGGGCCCGCGCCCGCCGAAGACACCCTCGAACTCTGTCT
GAAGATTGTAGTCTGAGTGAAAATATAAATTATTTAAAACTTTCAACAACGG
ATCTCTTGGTTCCGGCATCGATGAAGAACGCAGCGAAATGCGATACGTAAT
GTGAATTGCAAATTCAGTGAATCATCGAGTCTTTGAACGCACATTGCGCCC
CCTGGTATTCCGGGGGGCATGCCTGTCCGAGCGTCATTTCTGCCCTCAAGC
ACGGCTTGTGTGTTGGGCCCCGTCCTCCGATCCCGGGGGACGGGCCCGAA
AGGCAGCGGCGGCACCGCGTCCGGTCCTCGAGCGTATGGGGCTTTGTCAC
CCGCTCTGTAGGCCCGGCCGGCGCTTGCCGATCAACCCAAATTTTTATCCA
GGTTGACCTCGGATCAGGTAGGGATACCC

Figure S36. Gene sequences of *Penicillium* sp. QM-4

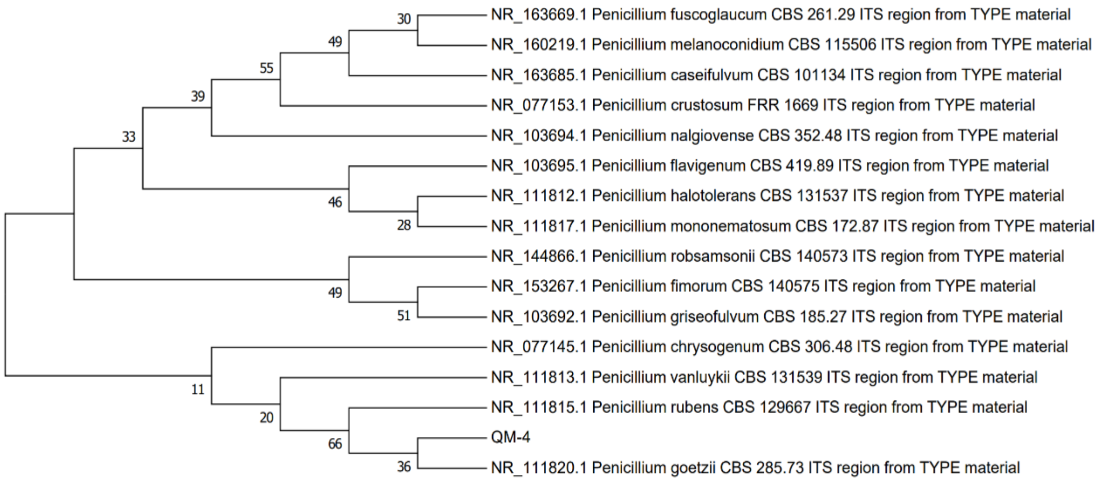

Figure S37. Phylogenetic tree of *Penicillium* sp. QM-4
